# Control of cardiac contractions using Cre-lox and degron strategies in zebrafish

**DOI:** 10.1073/pnas.2309842121

**Published:** 2024-01-09

**Authors:** Thomas Juan, Maëlle Bellec, Bárbara Cardoso, Héloïse Athéa, Nana Fukuda, Marga Albu, Stefan Günther, Mario Looso, Didier Y. R. Stainier

**Affiliations:** ^a^Department of Developmental Genetics, Max Planck Institute for Heart and Lung Research, Bad Nauheim 61231, Germany; ^b^German Centre for Cardiovascular Research (Deutsches Zentrum für Herz- Kreislaufforschung), Bad Nauheim 61231, Germany; ^c^Cardio-Pulmonary Institute, Bad Nauheim 61231, Germany; ^d^Bioinformatics and Deep Sequencing Platform, Max Planck Institute for Heart and Lung Research, Bad Nauheim 61231, Germany; ^e^Bioinformatics Core Unit, Max Planck Institute for Heart and Lung Research, Bad Nauheim 61231, Germany

**Keywords:** cardiac contractions, cardiac troponin T, Cre-lox, Degron, cpFRB2-FKBP

## Abstract

Cardiac contractions and hemodynamic forces are essential for organ development and homeostasis. Control over cardiac contractions can be achieved pharmacologically or optogenetically. However, these approaches lack specificity or require direct access to the heart. Here, we compare two genetic approaches to control cardiac contractions by modulating the levels of the essential sarcomeric protein Tnnt2a in zebrafish. We first recombine a newly generated *tnnt2a* floxed allele using multiple lines expressing Cre under the control of cardiomyocyte-specific promoters, and show that it does not recapitulate the *tnnt2a/silent heart* mutant phenotype in embryos. We show that this lack of early cardiac contraction defects is due, at least in part, to the long half-life of *tnnt2a* mRNA, which masks the gene deletion effects until the early larval stages. We then generate an endogenous Tnnt2a-eGFP fusion line that we use together with the zGRAD system to efficiently degrade Tnnt2a in all cardiomyocytes. Using single-cell transcriptomics, we find that Tnnt2a depletion leads to cardiac phenotypes similar to those observed in *tnnt2a* mutants, with a loss of blood and pericardial flow-dependent cell types. Furthermore, we achieve conditional degradation of Tnnt2a-eGFP by splitting the zGRAD protein into two fragments that, when combined with the cpFRB2-FKBP system, can be reassembled upon rapamycin treatment. Thus, this Tnnt2a degradation line enables non-invasive control of cardiac contractions with high spatial and temporal specificity and will help further understand how they shape organ development and homeostasis.

## Significance

Controlling cardiac contractions is critical to understand the role of hemodynamic forces during organ development and homeostasis. Existing pharmacological and optogenetic methods lack precision or require invasive procedures to prevent cardiac contractions during an extended period. The inactivation of the cardiac-specific sarcomeric protein Tnnt2a blocks cardiac contractions. Our study compares two genetic approaches to modulate Tnnt2a protein levels and thereby control cardiac contractions. We report that a novel degron-based strategy is superior to the Cre-lox system to induce Tnnt2a depletion and early cardiac contraction defects. This Tnnt2a degradation approach provides a non-invasive control of cardiac contractions with high spatial and temporal specificity, providing a critical tool to gain further insights into the mechanisms underlying organ development and homeostasis.

Cardiac contractions, which propel blood throughout the organism, are essential for animal development as well as the maintenance of physiological processes throughout the organism’s lifespan. Hemodynamic forces generated by blood flow are required to shape various aspects of organogenesis and tissue formation including endothelial cell differentiation and remodeling during heart and kidney morphogenesis (1–4). Current approaches to control cardiac contractions and blood flow include pharmacological treatments (5, 6), optogenetic switches (7), and global genetic loss-of-function conditions (5, 6). However, these methods lack precise spatiotemporal specificity or require constant access to the heart.

The zebrafish model has been used extensively to look at the role of cardiac contractions/blood flow in organ development (4, 5) because its external fertilization allows easy access to the embryos and larvae. In addition, the small size of their progeny allows their survival for an extended period of time in the absence of cardiac contractions/blood flow (4, 5).

Cardiac troponin T (TNNT2), a thin filament component of the sarcomere, is critical for heart function, as reduced expression of TNNT2 leads to structural defects in the sarcomeres (8). Zebrafish *tnnt2a* mutant and morphant embryos exhibit cardiomyopathy phenotypes similar to those observed in human TNNT2 patients (5, 9). They display a complete lack of cardiac contractions and fail to assemble sarcomeres, resulting in chamber enlargement (9), lack of trabeculation (10, 11), and lack of valves (12). Although Tnnt2a function, and therefore cardiac contractions, can be restored using overexpression constructs (9, 13), no tools are currently available to control Tnnt2 endogenous protein levels temporally and in specific subpopulations of cardiomyocytes.

Modulating RNA and protein expression in vivo can be achieved using multiple genetic tools (14, 15). However, the efficiency of conditional technologies that target DNA and RNA depends upon the targeted protein’s half-life, which is limiting when proteins have already been translated and/or are prone to transcriptional adaptation (16). To overcome these limitations, several protein targeting methods that rely on endogenous degradation pathways have been developed including one driven by a motif on the protein of interest (POI) called a degron (17). In zebrafish, the zGRAD degron system (18), adapted from the *Drosophila* deGradFP system (19), is used to target POIs tagged with a GFP-based fluorescent protein for degradation, using an anti-GFP nanobody fused to an F-box protein. The degradation of the POI is mediated through polyubiquitination and the proteasome. However, these approaches have not been tested on many endogenous targets in vertebrates (20, 21) and notably not on proteins integrated into tight subcellular structures such as the sarcomere.

To investigate the consequences of the loss of cardiac contractions in different cardiomyocyte populations and subsequent blood flow defects, we compare here two conditional loss-of-function tools targeting Tnnt2a at the DNA and protein levels. We generated a *tnnt2a* floxed allele, which recapitulates the *tnnt2a* mutant phenotype when recombined globally. However, we find that its recombination by Cre expressed under the control of cardiomyocyte-specific promoters does not affect embryonic heart function: If recombination occurs after gene transcription/translation has started, the existing mRNA and protein can mask the effects of the lack of the gene for at least 4 d. To circumvent this issue, we integrated an enhanced green fluorescent protein (eGFP) in the endogenous *tnnt2a* locus via CRISPR/Cas9-mediated genome editing and used it as a degron tag in conjunction with the zGRAD system. We show that Tnnt2a-eGFP protein degradation under the control of a cardiomyocyte-specific promoter leads to an embryonic phenotype similar to that of the *tnnt2a* mutant using morphological and single-cell transcriptomic analyses. Moreover, we improved the zGRAD system to make it rapamycin-inducible by fusing it with the induced heterodimer components cpFRB2 and FKBP. Taken together, our findings show that early sarcomeric-related phenotypes, otherwise not detectable using the Cre-Lox system, can be triggered using a degron system, thereby providing a most valuable tool to modulate cardiac contractions/blood flow with high temporal and spatial control.

## Results

### Global Recombination of a floxed *tnnt2a* Allele Induces Cardiac Contraction Defects.

**Fig. 1.**
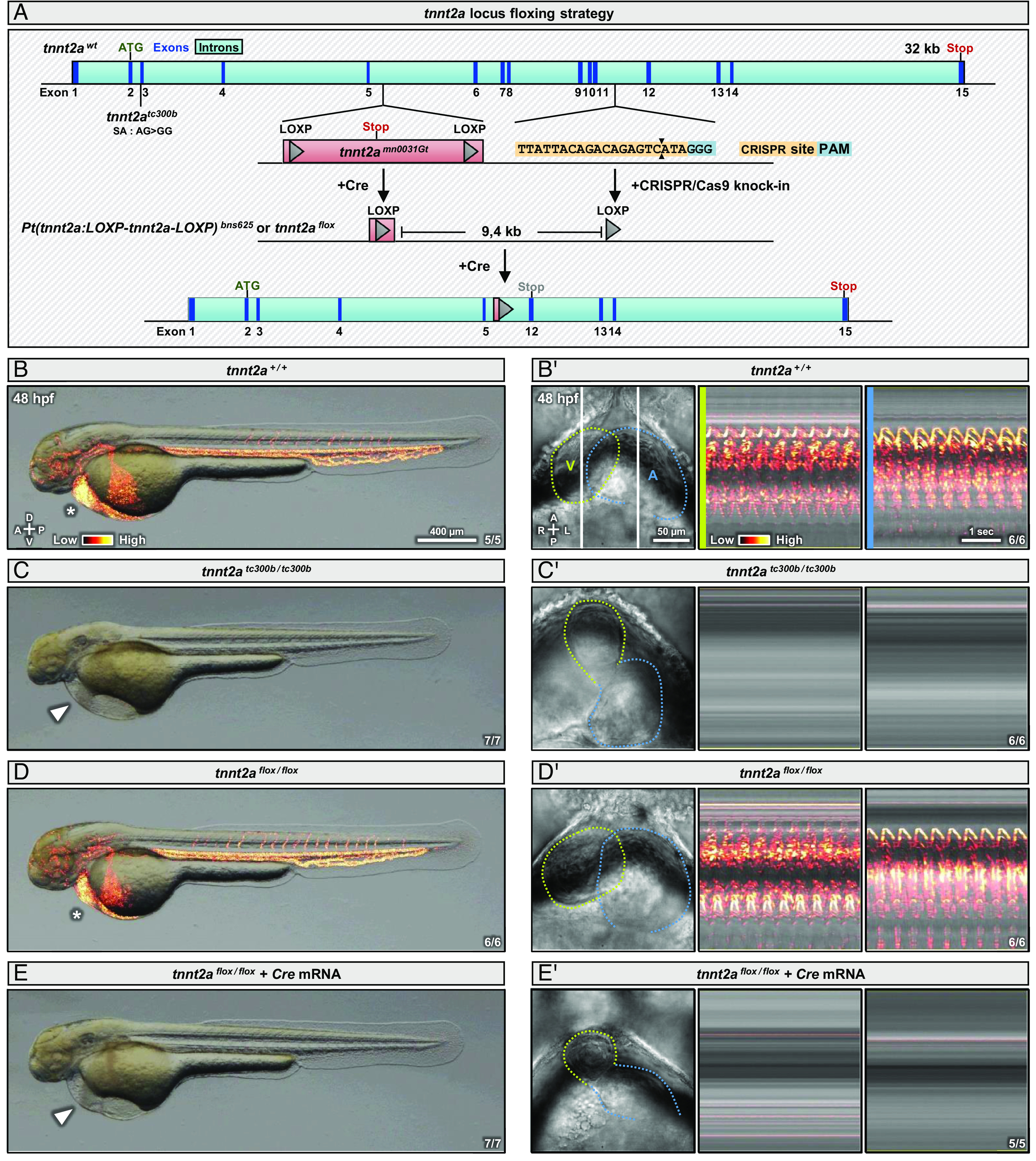
Global recombination of a floxed *tnnt2a* allele recapitulates the *tnnt2a* mutant phenotype. (*A*) Schematics of the *tnnt2a* locus showing the positions of the *tc300b* lesion and of the *mn0031* gene-trap cassette; the first LOXP was obtained through Cre recombination of the gene-trap line, and the second LOXP was inserted via CRISPR/Cas9 knock-in using a ssODN donor in the recombined background; Cre-mediated recombination of the floxed allele leads to the removal of exons 6 to 11, and the formation of a PTC in exon 12; splice acceptor (SA); gray “Stop” is a PTC. (*B*–*E*) Brightfield images of 48 hpf WT (*B*), *tnnt2a* mutant (*C*), and *tnnt2a^flox/flox^* embryos non-injected (*D*) or injected at the one-cell stage with *Cre* mRNA (*E*); arrowheads and asterisks indicate respectively the presence and absence of pericardial edema. (*B'*–*E’* ) Brightfield images and kymographs of hearts from 48 hpf WT (*B’* ), *tnnt2a* mutant (*C’* ), and *tnnt2a^flox/flox^* embryos non-injected (*D’* ) or injected at the one-cell stage with *Cre* mRNA (*E’* ); green lines outline the ventricle (V), blue lines outline the atrium (A), and vertical white lines indicate the reference axis of the kymographs. “Red hot” lookup table coloring (from Low to High) highlights the SD (*B*–*E*) or 3D variance (*B’*–*E’* ). Diagrams indicate the anterior–posterior (A–P), dorsal–ventral (D–V), and left–right (L–R) axes.

Zebrafish *tnnt2a* mutants display a stereotypical lack of cardiac contractions and blood flow at all stages (9). To modulate Tnnt2a protein levels and therefore cardiomyocyte contractions, we first floxed the *tnnt2a* locus. To do so, we used a previously generated floxed gene-trap insertion in intron 5 of the *tnnt2a* locus (*mn0031Gt*) (22), which we removed by injecting *Cre* mRNA, as previously described for the *fleer* locus (23), resulting in the presence of a single LOXP site (Fig. 1*A*). We then inserted a second LOXP site in intron 11 of the *tnnt2a* locus using a CRISPR/Cas9 knock-in approach (23) (Fig. 1*A* and *SI Appendix*, Fig. S1*A*), resulting in a premature termination codon (PTC) in exon 12 upon Cre recombination (Fig. 1*A* and *SI Appendix*, Fig. S1 *B* and *C*) and nonsense-mediated mRNA decay (NMD) (24) of the transcript (*SI Appendix*, Fig. S1*D*). We refer to the obtained *Pt(tnnt2a:LOXP-tnnt2a-LOXP)^bns625^* allele as *tnnt2a^flox^* hereafter. We find that *tnnt2a^flox/flox^* animals display a wild-type (WT)-like phenotype at 48 hours post-fertilization (hpf), with unaffected cardiac contractions or blood flow (Fig. 1 *B*, *B'*, *D*, and *D'* ). However, when injected with *Cre* recombinase mRNA, *tnnt2a^flox/flox^* embryos fully recapitulate the *tnnt2a^tc300b^* (9) mutant phenotype, with a complete lack of cardiac contractions (Fig. 1 *C'* and *E'* ) and blood flow (Fig. 1 *C* and *E*). Altogether, these data validate the *tnnt2a^flox^* allele as a productive conditional allele for Cre-lox-mediated recombination.

### Cardiomyocyte-Specific *tnnt2a* Knockout Fails to Recapitulate the *tnnt2a* Mutant Phenotype.

**Fig. 2.**
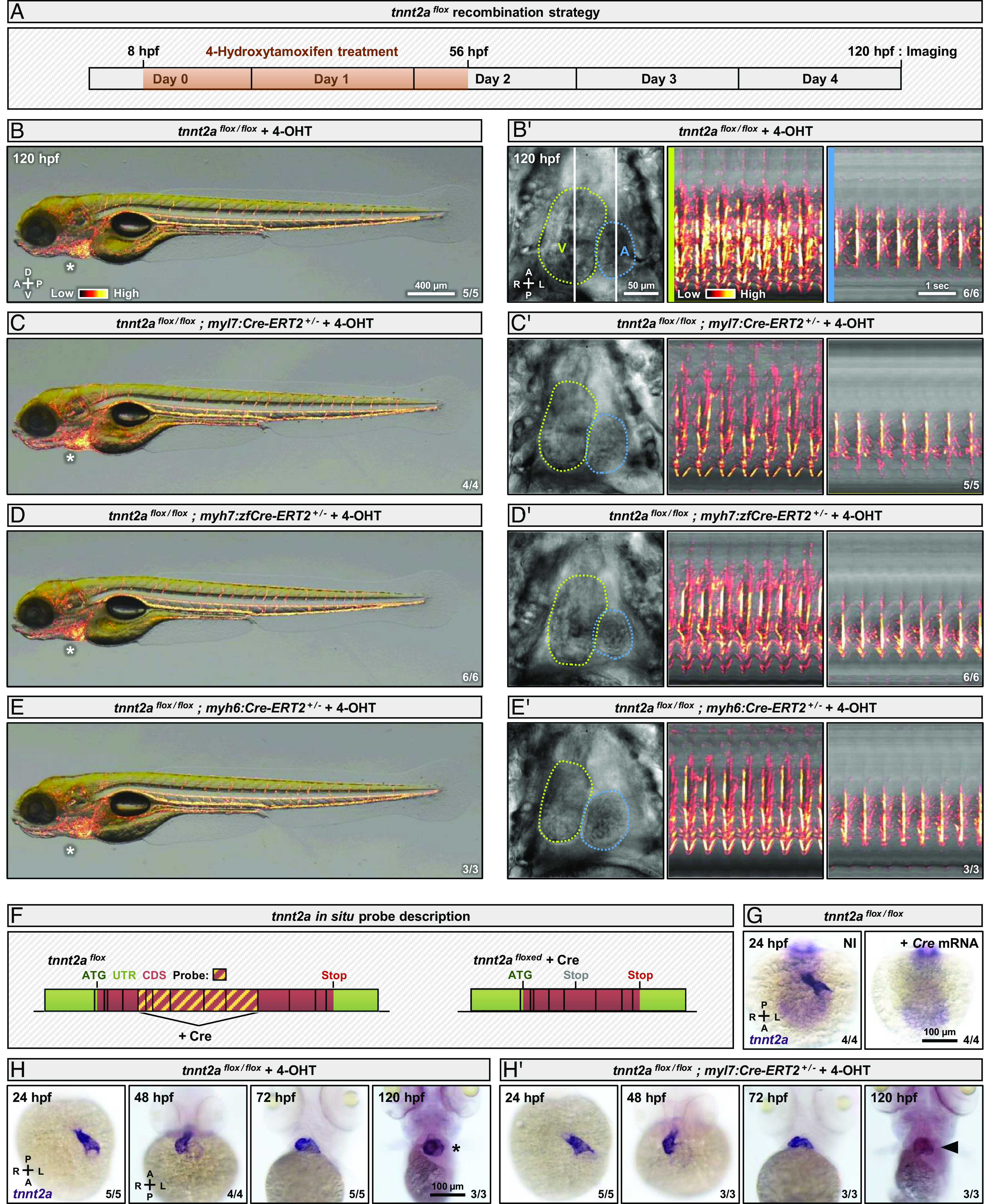
Myocardial Cre-lox-mediated *tnnt2a* deletion fails to induce early cardiac contraction defects. (*A*) Schematic of *tnnt2a* recombination strategy with 4-OHT treatment between 8 and 56 hpf to trigger early Cre-ERT2 activation, and imaging at 120 hpf. (*B*–*E*) Brightfield images of 120 hpf *tnnt2a^flox/flox^* (*B*), *tnnt2a^flox/flox^*; *myl7:Cre-ERT2^+/−^* (*C*), *tnnt2a^flox/flox^*; *myh7:zfCre-ERT2^+/−^* (*D*), and *tnnt2a^flox/flox^*; *myh6:Cre-ERT2^+/−^* (*E*) larvae treated with 4-OHT; asterisks indicate the absence of pericardial edema. (*B'*–*E’* ) Brightfield images and kymographs of hearts from 120 hpf *tnnt2a^flox/flox^* (*B’* ), *tnnt2a^flox/flox^*; *myl7:Cre-ERT2^+/−^* (*C’* ), *tnnt2a^flox/flox^*; *myh7:zfCre-ERT2^+/−^* (*D’* ), and *tnnt2a^flox/flox^*; *myh6:Cre-ERT2^+/−^* (*E’* ) larvae treated with 4-OHT; green lines outline the ventricle (V), blue lines outline the atrium (A), and vertical white lines indicate the reference axis of the kymographs. (*F*) Schematics of WT and *tnnt2a^flox^* mRNA showing that the region bound by the in situ hybridization probe is removed by Cre recombination; gray “stop” is a PTC. (*G*) Brightfield images of 24 hpf *tnnt2a^flox/flox^* embryos, non-injected or injected at the one-cell stage with *Cre* mRNA, and stained for *tnnt2a* expression. (*H* and *H’* ) Brightfield images of 24, 48, 72, and 120 hpf *tnnt2a^flox/flox^* (*H*), and *tnnt2a^flox/flox^*; *myl7:Cre-ERT2^+/−^* (*H’* ) embryos and larvae treated with 4-OHT, and stained for *tnnt2a* expression; asterisk and arrowhead indicate respectively WT and decreased *tnnt2a* mRNA levels in the heart. “Red hot” lookup table coloring (from Low to High) highlights the SD (*B*–*E* ) or 3D variance (*B’*–*E’*). Diagrams indicate the anterior–posterior (A–P), dorsal–ventral (D–V), and left–right (L–R) axes.

Robust Cre expression is required to mediate efficient recombination of endogenous floxed alleles in zebrafish (23, 25–29). Here, we used multiple transgenic lines expressing tamoxifen-inducible Cre-ERT2 (Fig. 2 *C*–*E'* ) or constitutive Cre (*SI Appendix*, Fig. S2 *A* and *A'* ) recombinases in cardiomyocytes under the control of *myl7* (30, 31) (Fig. 2 *C* and *C'* and *SI Appendix*, Fig. S2 *A* and *A’* ), *myh7/vmhc* (32) (Fig. 2 *D* and *D'* ), or *myh6/amhc* (33) (Fig. 2 *E* and *E'*) promoters. We treated the *Cre-ERT2* transgenic lines with 4-hydroxytamoxifen (4-OHT) from 8 to 56 hpf (Fig. 2*A*) to optimize early Cre activity in cardiomyocytes before 14 hpf, the time when *tnnt2a* starts to be expressed (34), and observed no cardiac contraction phenotype, or blood flow phenotype, up to 120 hpf (Fig. 2 *B*–*E'* and *SI Appendix*, Fig. S2 *A* and *A’* ). We then designed an in situ hybridization probe that spans the region of the floxed *tnnt2a* mRNA that is removed by Cre recombination (Fig. 2*F*) and validated its specificity at 24 hpf (Fig. 2*G*). We found that intact *tnnt2a* mRNA was still present at 24, 48, and 72 hpf in *tnnt2a^flox/flox^* embryos expressing *Cre-ERT2* in cardiomyocytes and treated with 4-OHT (Fig. 2 *H* and *H'* ) or expressing constitutive Cre in cardiomyocytes (*SI Appendix*, Fig. S2 *B* and *B'* ) and that at 120 hpf it appeared to be lower (Fig. 2 *H* and *H'* and *SI Appendix*, Fig. S2 *B* and *B’* ). These data indicate that the *tnnt2a^flox^* allele was recombined after its transcription had started. To further test this hypothesis, we assessed the recombination of the *tnnt2a^flox^* allele when Cre was constitutively expressed in cardiomyocytes and found that it was only visible at 48 hpf (*SI Appendix*, Fig. S2*C*). We then crossed the *myl7:Cre* (*SI Appendix*, Fig. S2*D*), *myl7:Cre-ERT2* (*SI Appendix*, Fig. S2*E*), *myh7:zfCre-ERT2* (*SI Appendix*, Fig. S2*F*), and *myh6:Cre-ERT2* (*SI Appendix*, Fig. S2*G*) lines with a *ubb:LOXP-eGFP-LOXP-mCherry* line, treated the *Cre-ERT2* lines with 4-OHT, and assessed mCherry expression, which appears upon deletion of the eGFP cassette. We found that no mCherry expression could be detected at 24 hpf (*SI Appendix*, Fig. S2 *D*–*G*), a time when *tnnt2a* mRNA is already abundant (Fig. 2*G*), in contrast to clear mCherry expression at 48 hpf (*SI Appendix*, Fig. S2 *D*–*G*). Moreover, we activated *tnnt2a^flox^* recombination at 6 and 24 hpf using a *hsp70l:Cre* line (*SI Appendix*, Fig. S3*A*) and validated its functionality by in situ hybridization (*SI Appendix*, Fig. S3*B*). We observed no cardiac contraction or blood flow phenotype up to 120 hpf when Cre expression was induced by heat shock at 24 hpf, but when Cre expression was induced at 6 hpf, we observed the *tnnt2a* mutant phenotype (*SI Appendix*, Fig. S3 *C*–*E'* ). Together, these observations suggest that Cre-mediated recombination of *tnnt2a^flox^* using cardiomyocyte promoters occurs too late to produce an early cardiac phenotype.

### Functional *eGFP* Integration into the *tnnt2a* Locus.

**Fig. 3.**
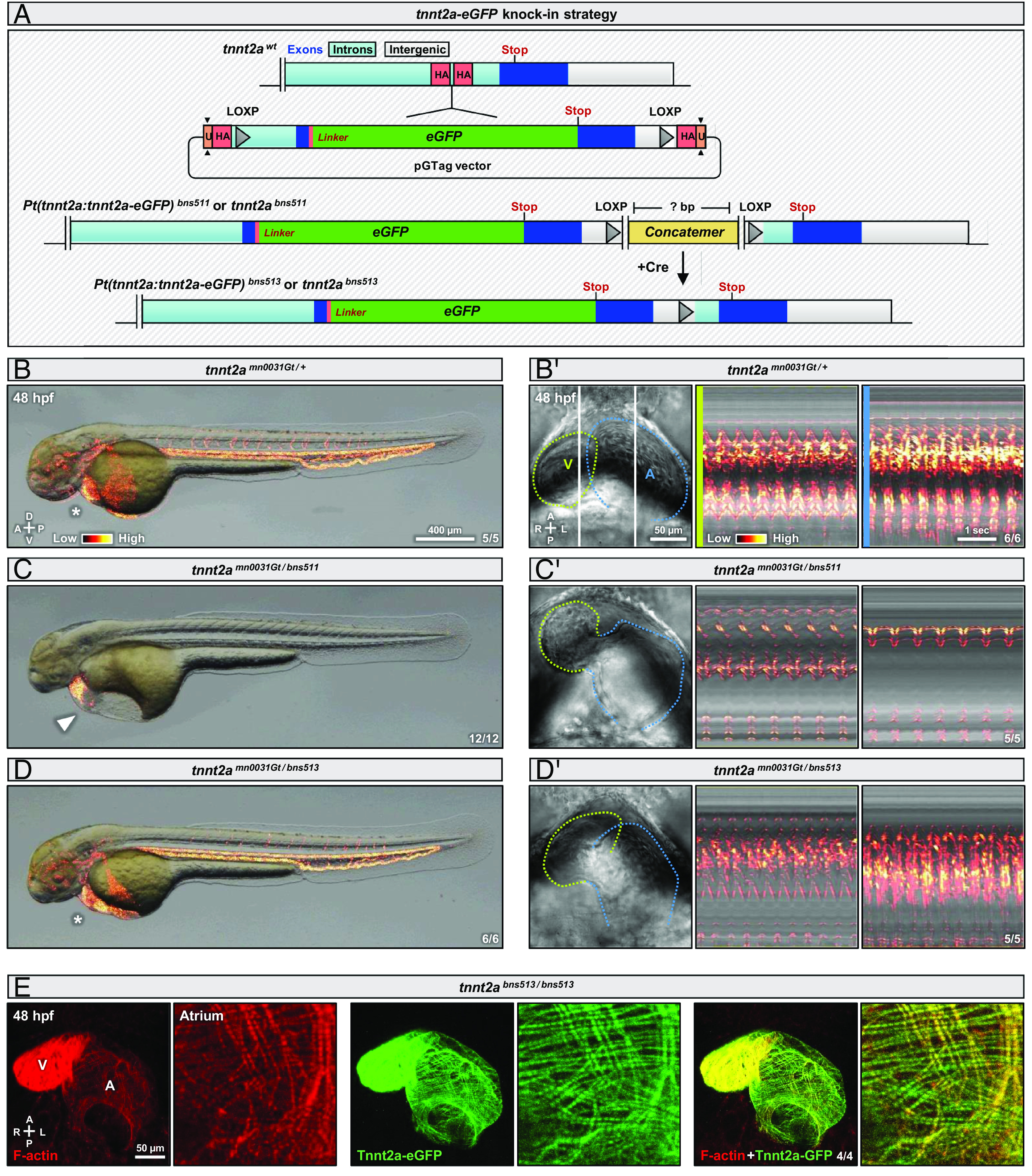
Viable endogenous eGFP-tagging recapitulates the Tnnt2a expression pattern. (*A*) Schematics of *tnnt2a* locus showing the insertion of an eGFP cassette at the C-terminus of the protein using the CRISPR/Cas9 knock-in vector pGTag; the knock-in cassette contains the last exon of *tnnt2a* and an eGFP before the stop codon, separated by a GSSS linker, and it is inserted in the last intron; 48 bp homology arms (HA) flanking the Cas9 cleavage site were used and the vector is cleaved at a universal (U) CRISPR site; the *tnnt2a^bns511^* allele contains a concatemer composed of multiple insert and vector backbone copies, which was removed by injecting *Cre* mRNA at the one-cell stage to create the *tnnt2a^bns513^* allele. (*B*–*D*) Brightfield images of 48 hpf *tnnt2a^mn0031Gt/+^* (*B*), *tnnt2a^mn0031Gt/bns511^* (*C*), and *tnnt2a^mn0031Gt/bns513^* (*D*) embryos; arrowhead and asterisks indicate, respectively, the presence and absence of pericardial edema. (*B'*–*D'*) Brightfield images and kymographs of hearts from 48 hpf *tnnt2a^mn0031Gt/+^* (*B’* ), *tnnt2a^mn0031Gt/bns511^* (*C’* ), and *tnnt2a^mn0031Gt/bns513^* (*D’ *) embryos; green lines outline the ventricle (V), blue lines outline the atrium (A), and vertical white lines indicate the reference axis of the kymographs. (*E*) Confocal image of a heart from a 48 hpf *tnnt2a^bns513/bns513^* embryo stained for F-actin with Phalloidin; maximum z-projection; annotations correspond to the ventricle (V) and atrium (A). “Red hot” lookup table coloring (from Low to High) highlights the SD (*B*–*D*) or 3D variance (*B’*–*D’* ). Diagrams indicate the anterior–posterior (A–P), dorsal–ventral (D–V), and left–right (L–R) axes.

Protein degradation methods have been reported to give access to phenotypes otherwise not visible using conventional DNA or RNA targeting tools (14). We thus investigated whether Tnnt2a protein degradation could give rise to early cardiac phenotypes, in contrast to the Cre-lox system. To do so, we used the zGRAD system which requires the targeted protein to be tagged with a GFP-based fluorescent protein (18). We introduced in the last intron of *tnnt2a* a floxed version of the last exon of *tnnt2a* with an *eGFP* cloned upstream of the stop codon, separated by a flexible linker (35), using the GeneWeld method (36) (Fig. 3*A*). We found a carrier (*bns511*) for this eGFP insertion, albeit one with a concatemer consisting of multiple vector backbones and inserts (Fig. 3*A* and *SI Appendix*, Fig. S4 *A* and *B*). The evaluation of the concatemer structure by sequencing revealed that it was flanked by two LOXP sites, which we used to excise the concatemer by injecting *Cre* mRNA, thereby creating the *bns513* allele (Fig. 3*A* and *SI Appendix*, Fig. S4 *A* and *B*) and we refer to the obtained *Pt(tnnt2a:tnnt2a-eGFP)^bns511 and bns513^* alleles as *tnnt2a^bns511 and bns513^* hereafter. We found that *tnnt2a^bns511^* is a hypomorphic allele that displays decreased cardiac contractions and blood flow when crossed with the strong mutant allele *tnnt2a^mn0031Gt^* (Fig. 3 *B*–*C'* ) and that removal of the concatemer in *tnnt2a^bns513^* rescued these phenotypes (Fig. 3 *D* and *D'* ). We further observed that the concatemer-containing *tnnt2a^bns511^* allele, although producing the same *tnnt2a-eGFP* mRNA as *tnnt2a^bns513^* does (*SI Appendix*, Fig. S4*C*), exhibits a decrease in *tnnt2a* mRNA levels, which is partially rescued in *tnnt2a^bns513^* (*SI Appendix*, Fig. S4*D*). We thus kept *tnnt2a^bns513^* for our analyses and show that the eGFP in this allele is expressed in the heart and is integrated into the sarcomeres (Fig. 3*E*). Taken together, our data show that the eGFP tagging of Tnnt2a at its C-terminus produces a functional protein, and one that recapitulates the Tnnt2a expression pattern (13, 22, 34).

### Tnnt2a-eGFP Protein Degradation Recapitulates the *tnnt2a* Mutant Phenotype.

**Fig. 4.**
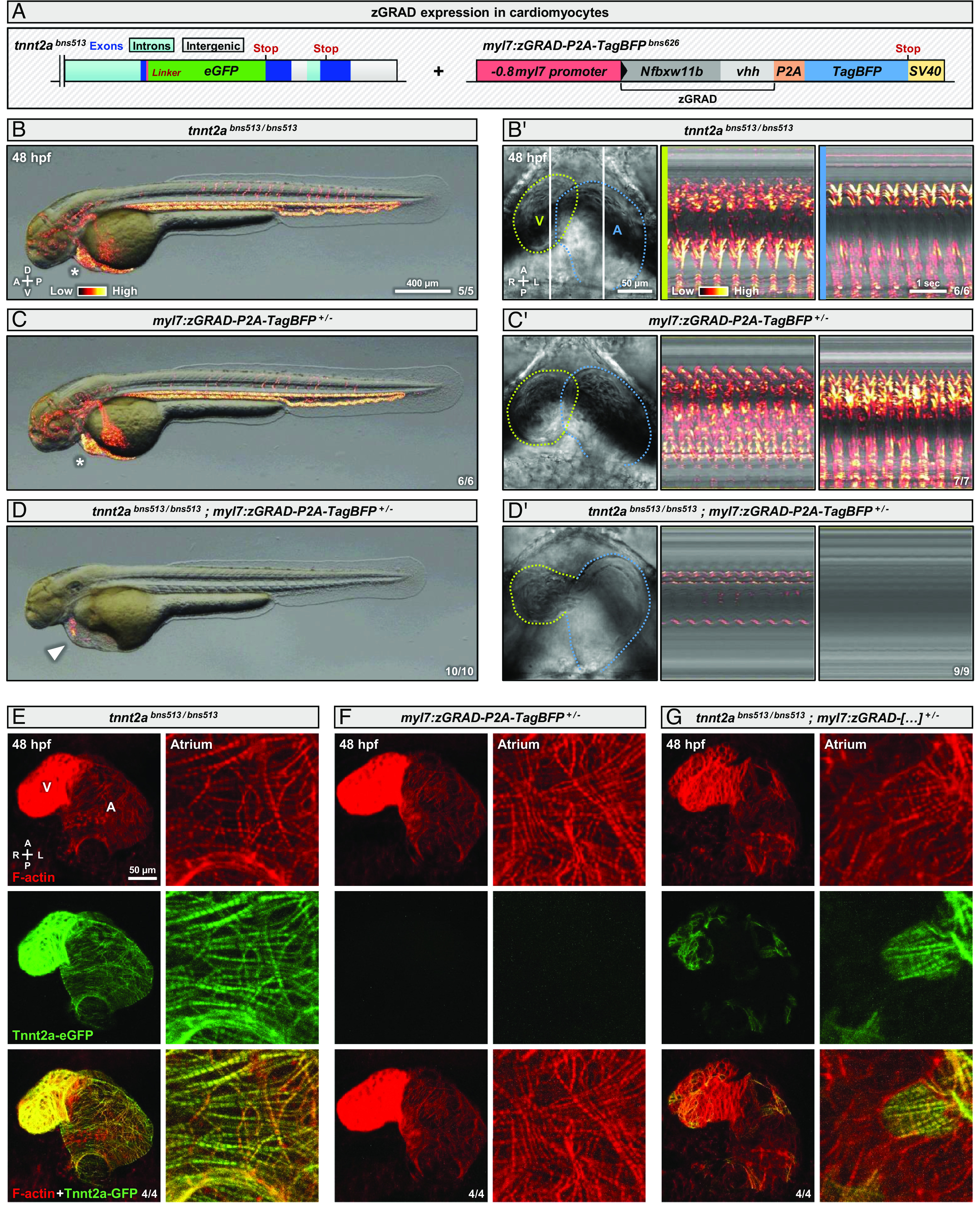
Myocardial Tnnt2a-eGFP degradation recapitulates the *tnnt2a* mutant phenotype. (*A*) Schematics of *tnnt2a* locus showing the *eGFP* insertion at the C-terminus of the protein in the *tnnt2a^bns513^* allele and a tol2-generated allele containing the zGRAD system with a TagBFP reporter separated by a P2A peptide under the control of the myocardial *myl7* promoter; the zGRAD transgene is composed of the N-terminus of the F-box and WD repeat domain containing 11b (Nfbxw11b) protein and an anti-GFP nanobody (vhh). (*B*–*D*) Brightfield images of 48 hpf *tnnt2a^bns513/bns513^* (*B*), *myl7:zGRAD-P2A-TagBFP^+/-^* (*C*), and *tnnt2a^bns513/bns513^*; *myl7:zGRAD-P2A-TagBFP^+/−^* (*D*) embryos; arrowhead and asterisks indicate, respectively, the presence and absence of pericardial edema. (*B'*–*D’* ) Brightfield images and kymographs of hearts from 48 hpf *tnnt2a^bns513/bns513^* (*B’* ), *myl7:zGRAD-P2A-TagBFP^+/−^* (*C’* ), and *tnnt2a^bns513/bns513^*; *myl7:zGRAD-P2A-TagBFP^+/−^* (*D’* ) embryos; green lines outline the ventricle (V), blue lines outline the atrium (A), and vertical white lines indicate the reference axis of the kymographs. (*E*–*G*) Confocal image of hearts from 48 hpf *tnnt2a^bns513/bns513^* (*E*), *myl7:zGRAD-P2A-TagBFP^+/−^* (*F*), and *tnnt2a^bns513/bns513^*; *myl7:zGRAD-P2A-TagBFP^+/−^* (*G*) embryos stained for F-actin with Phalloidin; maximum z-projection; annotations correspond to the ventricle (V) and atrium (A). “Red hot” lookup table coloring (from Low to High) highlights the SD (*B*–*D*) or 3D variance (*B’*–*D’*). Diagrams indicate the anterior–posterior (A–P), dorsal–ventral (D–V), and left–right (L–R) axes.

The efficiency of the zGRAD system correlates with its expression levels (18, 20). Moreover, it was reported that *tnnt2a* is expressed at high levels during embryogenesis compared to other genes that have been targeted for protein degradation using the zGRAD system, such as *cxcr4b* and *vangl2* (18, 20, 37). Thus, we built a construct and established a transgenic line expressing the zGRAD system under the control of the strong myocardial *myl7* promoter (38) (Fig. 4*A*). We found that 48 hpf *tnnt2a^bns513/bns513^; myl7:zGRAD-P2A-TagBFP^+/−^* (which we also refer to as Tnnt2a degron) embryos display strong cardiac contraction defects (Fig. 4*D'* ) and lack blood flow (Fig. 4*D*), reminiscent of *tnnt2a* mutants (Fig. 1 *C* and *C'* ), compared with controls (Fig. 4 *B*–*C'* ). The analysis of Tnnt2a-eGFP expression in Tnnt2a degron embryos shows that the fluorescent protein has been strongly degraded by 48 hpf (Fig. 4*G*), compared with controls (Fig. 4 *E* and *F*), and that Tnnt2a-eGFP protein degradation leads to sarcomere disassembly (Fig. 4 *E*–*G*), similar to what is observed in *tnnt2a* mutants (9). The degradation is not only restricted to mature Tnnt2a-eGFP protein but affects all forms of the protein (*SI Appendix*, Fig. S5*A*). Furthermore, we show that the degradation is visible at earlier stages including at 24 and 36 hpf (*SI Appendix*, Fig. S5 *B*–*D*). Together, these observations show that the sarcomeric protein Tnnt2a can be targeted to proteasomal degradation and trigger a *tnnt2a* mutant-like phenotype in embryos, in contrast to the Cre-lox system (Fig. 2).

### The Transcriptomes of Tnnt2a Degron and *tnnt2a* Mutant Hearts Appear Similar at the Single-Cell Level.

**Fig. 5.**
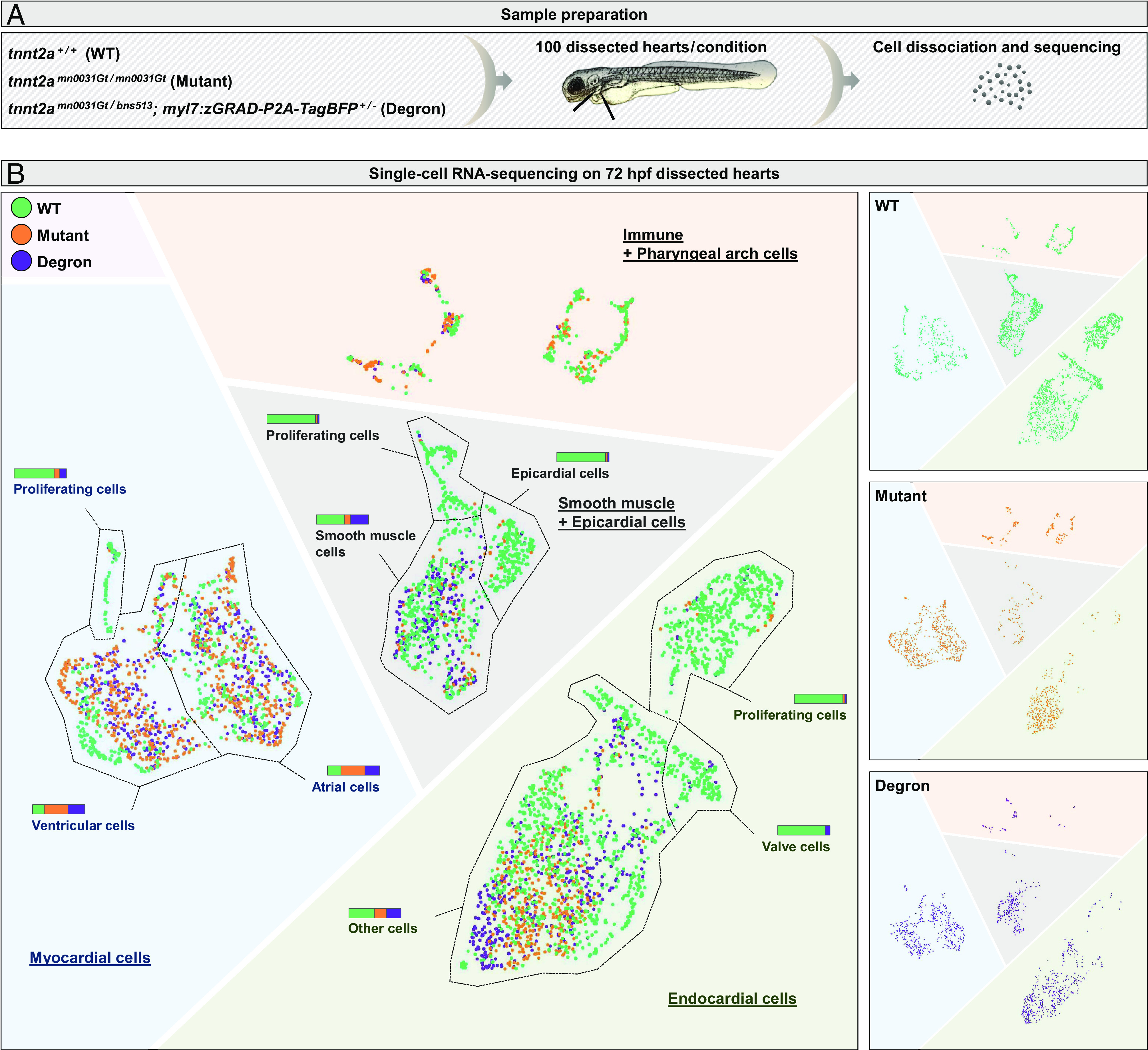
The cardiac transcriptomes of Tnnt2a degrons and *tnnt2a* mutants display high similarity at the single cell level. (*A*) Schematic and description of the sample preparation for single-cell RNA-sequencing; 100 hearts from 72 hpf WT, *tnnt2a^mn0031Gt/mn0031Gt^* (Mutant), and *tnnt2a^mn0031Gt/bns513^*; *myl7:zGRAD-P2A-TagBFP^+/−^* (Degron) larvae were dissected, dissociated and the single cell suspension sequenced. (*B*) Uniform manifold approximation and projection (UMAP) representation of the data; stacked columns represent the numbers of WT (green), mutant (orange), and degron (violet) cells for each annotated population; UMAP is displayed merged (*Left*) or split by genotype (*Right*).

Although conditional protein degradation tools can trigger mutant-like phenotypes, it remains unclear how similar they are at the single-cell level. Notably, *tnnt2a* is expressed in other cardiac tissues than the myocardium, where Tnnt2a degradation takes place in our model, such as the outflow tract smooth muscle tissue (13). Moreover, the removal of Tnnt2a after sarcomeric assembly could have a different consequence compared to the absence of Tnnt2a. To investigate whether the cardiac morphogenetic changes associated with the loss of cardiac contractions and blood flow in *tnnt2a^mn0031Gt^* mutants are also present in Tnnt2a degron animals, we performed single-cell RNA-sequencing on dissected hearts (Fig. 5 *A* and *B*). We selected 72 hpf to carry out this analysis, as several flow-dependent cardiac structures are visible at this stage including epicardial coverage (39), elongated valves (40), and trabeculae (10). We identified four main cell populations, myocardial cells, endothelial cells, smooth muscle, and epicardial cells, as well as immune and pharyngeal arch cells (Fig. 5*B* and *SI Appendix*, Fig. S6 *A* and *B*), which we annotated based on published in situ hybridization data (The Zebrafish Information Network, ZFIN). We found that the *tnnt2a* mutant and Tnnt2a degron cells cluster together compared with the WT sibling cells (Fig. 5*B* and *SI Appendix*, Fig. S6 *A*–*F*). As expected, *tnnt2a* is expressed in myocardial and smooth muscle cells and its levels are reduced but still detectable in *tnnt2a* mutants, possibly due to alternative splicing over the gene-trap cassette (22) (*SI Appendix*, Fig. S7*A*). More surprisingly, *tnnt2a*, but not Tnnt2a-eGFP, is also expressed in endocardial valve cells (*SI Appendix*, Fig. S7 *A* and *B*). Both *tnnt2a* mutants and Tnnt2a degrons display a lack of proliferating cells [i.e., *mki67+* (41)] (*SI Appendix*, Fig. S6 *B* and *C*), epicardial cells [i.e., *tcf21+* (42)] (*SI Appendix*, Fig. S6 *B* and *D*), and endocardial valve cells [i.e., *spp1+* (43)] (*SI Appendix*, Fig. S6 *B* and *E*); however, they exhibit an endocardial upregulation of *flt4* (*SI Appendix*, Fig. S6*F*), which is negatively regulated by blood flow (44). Moreover, we observed a higher number of myocardial cells and a lower number of smooth muscle cells in *tnnt2a* mutants and Tnnt2a degrons compared with wild types (*SI Appendix*, Fig. S6*B*). These data indicate that cardiac morphogenesis is affected similarly after Tnnt2a protein degradation as in *tnnt2a* mutants.

### Drug-Mediated Activation of the zGRAD System in Cardiomyocytes.

**Fig. 6.**
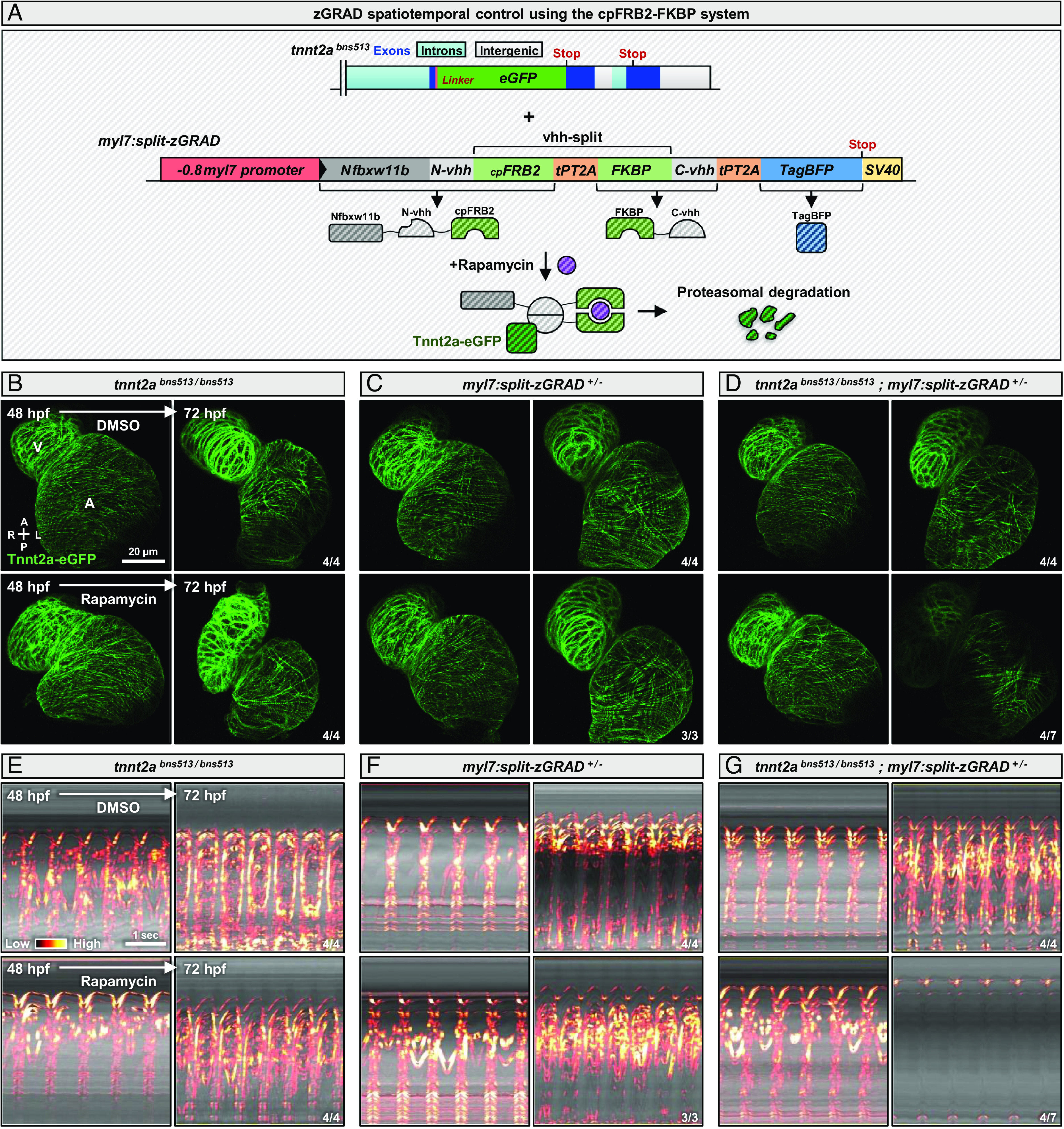
The cpFRB2-FKBP system enables fast temporal control of zGRAD activation. (*A*) Schematics of the *tnnt2a* locus showing the *eGFP* insertion at the C-terminus of the protein in the *tnnt2a^bns513^* allele and a tol2-generated allele containing the zGRAD elements split by the cpFRB2-FKBP system at the GFP nanobody region (split-zGRAD); upon rapamycin treatment, the cpFRB2 and FKBP proteins dimerize, reconstitute a functional GFP nanobody, and the zGRAD can target Tnnt2a-eGFP for proteasomal degradation. (*B*–*D*) Confocal images of hearts from 48 and 72 hpf *tnnt2a^bns513/bns513^* (*B*), *myl7:split-zGRAD^+/−^* (*C*); and *tnnt2a^bns513/bns513^*; *myl7:split-zGRAD^+/−^* (*D*) animals treated with DMSO or rapamycin from 48 to 72 hpf; maximum z-projection; annotations correspond to the ventricle (V) and atrium (A); diagram indicates the anterior–posterior (A–P) and left–right (L–R) axes. (*E*–*G*) kymographs of hearts from 48 and 72 hpf *tnnt2a^bns513/bns513^* (*E*), *myl7:split-zGRAD^+/−^* (*F*), and *tnnt2a^bns513/bns513^*; *myl7:split-zGRAD^+/−^* (*G*) animals treated with DMSO or rapamycin from 48 to 72 hpf; the reference axis of the kymograph is centered on the atrium; “Red hot” lookup table coloring (from Low to High) highlights the standard 3D variance. The same animals are used to image Tnnt2a-eGFP expression (*B*−*D*) and cardiac contractions (*E*−*G*).

The zGRAD system can be controlled temporally using genetic tools such as heat shock-responsive (18) or tamoxifen-inducible (20) promoters. To achieve temporal control in cardiomyocytes, we generated *tnnt2a^bns513/bns513^* animals carrying a *hsp70l:zGRAD-IRES-h2a-tagBFP* (18) or *actb2:LOXP-mCherry-LOXP-zGRAD* (20) transgene. We activated the *hsp70l* transgene by heat shock at 32 hpf (*SI Appendix*, Fig. S8 *A* and *B*) and the *actb2* transgene by removing the stop cassette in cardiomyocytes using a *myl7:Cre-ERT2* transgene together with 4-OHT treatment (*SI Appendix*, Fig. S8 *C* and *C'* ). We observed no degradation of Tnnt2a-eGFP at 48 hpf in either case (*SI Appendix*, Fig. S8 *B* and *C'* ), compared with control siblings (*SI Appendix*, Fig. S8 *A* and *C*). We hypothesized that this lack of degradation was due to low levels of zGRAD expression. To overcome this issue, we created a transgenic line expressing zGRAD under the control of the strong *myl7* promoter, activable by 4-OHT treatment after crossing with the *myl7:Cre-ERT2* line (*SI Appendix*, Fig. S8 *D* and *D'* ). However, we also failed to observe Tnnt2a-eGFP degradation in these animals (*SI Appendix*, Fig. S8*D*) compared with control siblings (*SI Appendix*, Fig. S8*D'* ). We previously showed that the *myl7:Cre-ERT2* line does not efficiently recombine LOXP sites during early cardiomyocyte development (i.e., at 24 hpf) (*SI Appendix*, Fig. S2 *D* and *E*), which could be an explanation for the weak zGRAD expression after Cre-ERT2 activation at 48 hpf (*SI Appendix*, Fig. S8 *C'* and *D'* ). To activate the zGRAD system post-translationally and without using Cre, we then turned to the cpFRB2-FKBP system (45) (Fig. 6*A*). We split the anti-GFP nanobody that is part of the zGRAD system using the *cpFRB2* and *FKBP* genes (45) separated by a tandem P2A-T2A peptide (tPT2A) for high-efficiency cleavage (46) (split-zGRAD). Upon rapamycin treatment, cpFRB2 and FKBP dimerize to reconstitute a functional anti-GFP nanobody (45) and therefore also potentially a functional zGRAD system (Fig. 6*A*). We injected at the one-cell stage DNA constructs containing the split-zGRAD system under the control of the myocardial *myl7* (38) (*SI Appendix*, Fig. S9*A*), *myh7* (32) (*SI Appendix*, Fig. S9*B*), and *myh6* (33) (*SI Appendix*, Fig. S9*C*) promoters. We found that the myocardial-positive cells displayed Tnnt2a-eGFP degradation after rapamycin treatment between 48 and 72 hpf (*SI Appendix*, Fig. S9 *A*–*C*), showing that Tnnt2a can be degraded when already integrated in the sarcomere. We also established a stable *myl7:split-zGRAD* transgenic line and found that the same rapamycin treatment induces Tnnt2a-eGFP degradation (Fig. 6 *B*–*D*) and cardiac contraction defects (Fig. 6 *E*–*G*) in the experimental larvae, without adverse effects on cardiac morphology or rhythmicity in the control larvae. These findings suggest that the cpFRB2-FKBP system can efficiently activate the zGRAD system and degrade eGFP.

## Discussion

Genetically encoded protein degradation tools can target a wide range of intracellular proteins, mimicking DNA and RNA loss-of-function phenotypes (14, 15). Although previous studies have shown the potential of these techniques to access phenotypes associated with proteins that have a long half-life (21), a comprehensive investigation of the conditional depletion of an endogenous protein in a vertebrate model, coupled with a systematic comparison to a conditional DNA targeting tool, has been lacking. In our study, we use CRISPR/Cas9 knock-in approaches and single-cell transcriptomics to show that the depletion of degron-tagged Tnnt2a proteins can trigger early cardiac phenotypes, in contrast to the Cre-lox controlled gene deletion system. Moreover, we improved the zGRAD protein degradation system by fusing it with the drug-inducible cpFRB2-FKBP heterodimer (45).

Cardiac contractions and hemodynamic forces are essential for heart and vascular morphogenesis, as well as the maintenance of their function in adults (2). However, the genetic control of contractions in specific subsets of cardiomyocytes remains challenging, particularly at later developmental stages where light-inducible systems face limitations (7). To address this challenge, we established a floxed *tnnt2a* allele to control cardiac contractions and blood flow using Cre recombinases, as lack of *tnnt2a* function causes the *silent heart* phenotype and sarcomere disassembly (9). We functionally validated the *tnnt2a* floxed allele using global recombination and showed that the deletion of exons 6 to 11 of *tnnt2a* leads to a PTC, protein truncation, and mRNA level reduction via NMD. Notably, expressing Cre using conventional myocardial drivers failed to recapitulate the *tnnt2a* mutant phenotype, as *tnnt2a* mRNA is already being expressed when the Cre became active, thereby masking gene loss during early development. The lack of early cardiac contraction phenotype could also be due to the long half-life of Tnnt2a protein, especially after incorporation into the sarcomeres. Moreover, it is also possible that the myocardial Cre drivers do not efficiently recombine the floxed allele at early stages, as suggested by the *hsp70l:Cre*-mediated recombination, which is not fully penetrant (*SI Appendix*, Fig. S3 *D* and *D'* ). Altogether, these data highlight the necessity to generate floxed alleles that report recombination (25, 27, 28).

As the Cre-lox system appeared unsuitable to trigger early Tnnt2a depletion, we turned to the zGRAD protein degradation system to control Tnnt2a levels in the heart and induce cardiac contraction defects. We generated an eGFP-tagged Tnnt2a line via CRISPR-Cas9 knock-in and retrieved a *tnnt2a* hypomorphic allele displaying a concatemer insertion consisting of multiple vector backbones and inserts, as previously observed (47). We showed that removal of the concatemer was sufficient to partially rescue *tnnt2a* mRNA levels, suggesting that it potentially interfered with *tnnt2a* transcription and/or mRNA stability. We then overexpressed zGRAD in myocardial cells and found that it can efficiently degrade Tnnt2a-eGFP and trigger *tnnt2a* mutant-like cardiac contraction and blood flow phenotypes. We hypothesize that the WT *tnnt2a* mRNA copies that are generated by alternative splicing in the *bns513* allele (*SI Appendix*, Fig. S4*C*) can account for the observed weak contractility in spite of the strong Tnnt2a-eGFP degradation detected by fluorescence and western blot analyses. Notably, we did not observe toxicity-related effects after zGRAD expression in myocardial cells, similar to previous reports on other tissues (18, 20), and in Tnnt2a-eGFP transgenic animals. It will be interesting to analyze the consequences on blood flow of Tnnt2a-eGFP degradation in other populations of myocardial cells using different drivers to control zGRAD expression (32, 33, 48, 49).

Although Tnnt2a degron and *tnnt2a* mutant embryos appear morphologically similar, we investigated whether it was also the case at the single-cell level using a transcriptomic approach. We found that both Tnnt2a degron and *tnnt2a* mutant hearts display a reduction in flow-responsive cell types such as epicardial cells (39), smooth muscle cells (50), and endocardial valve cells (12), as well as an upregulation of the flow-repressed gene *flt4* in endocardial cells (44). We also found that proliferation was not only inhibited in endocardial cells (51), but in all cardiac cell types, following loss of cardiac contractions/blood flow. However, the number of myocardial cells was higher in *tnnt2a* mutants and Tnnt2a degrons than in controls, possibly due to increased cardiac progenitor cell differentiation (52). For all the cell types, Tnnt2a degron hearts appear to display an intermediate number of cells between wild types and *tnnt2a* mutants, probably due to the fact that they still partially contract. Unexpectedly, we found that *tnnt2a* was also expressed in endocardial valve cells. It has been reported that genes thought to be restricted to myocardial cells can be expressed in endocardial cells (53). However, unlike the other markers expressed broadly in endocardial cells, *tnnt2a* expression was strongly enriched in valve cells. Whether this endocardial expression results in translation remained to be determined (53). Here, our data suggest that it is not the case as expression of the Tnnt2a-eGFP fusion protein was not observed in endocardial valve cells, but we cannot exclude that it could also be because Tnnt2a has a shorter half-life in these cells. Nevertheless, as *tnnt2a* deletion is one of the most commonly used methods to investigate the effect of blood-flow deprivation on endocardial valve formation (54, 55), future work should address whether Tnnt2a has a role in valve formation independently of its function in myocardial cells. Overall, we anticipate that further analysis of this cardiac contraction/blood flow-deprived dataset will contribute to the identification of new flow-responsive genes for several cardiac cell populations.

Among the degron-based protein degradation systems, the auxin-inducible degron has emerged as the leading cross-species tool (17, 21, 56–60). This system offers the advantage of being drug-inducible, allowing reversibility upon drug removal. Although initial work showed that it could be adapted for studies in zebrafish (61), it was later shown that the system exhibited leakiness without auxin (18). Therefore, we decided to use the alternative zGRAD system, which can lead to strong protein degradation but cannot be activated by drugs. We attempted to control zGRAD levels transcriptionally in myocardial cells using heat-shock-responsive and tamoxifen-inducible transgenes but observed no degradation of Tnnt2a-eGFP using these approaches. Subsequently, we adapted the induced heterodimer components cpFRB2 and FKBP to zGRAD and more precisely to the GFP nanobody that composes it. This approach has already been used successfully to split proteins such as Cre (62) and Cas9 (63), enabling their inducible activation via rapamycin treatment. We then achieved rapamycin-mediated degradation of Tnnt2a-eGFP in various populations of cardiomyocytes using this adapted split-zGRAD system. We observed that Tnnt2a-eGFP degradation was stronger after transient overexpression of the split-zGRAD construct compared with the stable line we generated. This observation highlights the fact that multiple transgene copies and a high expression level are necessary to reach high degradation efficiency. Future work could focus on adapting the cpFRB2-FKBP system to other small protein binders besides the anti-GFP nanobody, in order to target protein to degradation using smaller epitopes (64–66). Moreover, recent studies have shown that the leakiness and toxicity observed with the auxin-inducible degron system could be reduced (58, 67), suggesting that it could be used as an alternative protein degradation approach in zebrafish.

In humans, mutations in *TNNT2* are major causes of hypertrophic cardiomyopathy and dilated cardiomyopathy, which are diseases that affect the heart muscle, causing structural and functional cardiac abnormalities (8, 68, 69). *TNNT2* mutations often lead to alterations of, and decreased sensitivity to, cardiac Ca^2+^ levels, and a reduction of contractility (70). Therefore, controlling Tnnt2a protein levels, using the degron approach described here to mimic the loss of contractility observed in human patients could lead to new insights.

In summary, our findings indicate that the zGRAD approach is a superior cardiomyocyte-targeting system compared with the Cre-lox tool as evidenced by its ability to induce early cardiac phenotypes. Moreover, we suggest that the Tnnt2a degron system holds the potential for precise control of cardiac contractions and blood flow, opening new avenues to study the role of hemodynamic forces on organ development and homeostasis.

## Methods

### Zebrafish Husbandry.

Zebrafish husbandry was performed under institutional (MPG) and national (German) ethical and animal welfare regulations. Larvae were raised under standard conditions. Adult zebrafish were maintained in 3.5 L tanks at a stock density of 10 zebrafish/L with the following parameters: water temperature: 27 to 27.5 °C; light/dark cycle: 14/10; pH: 7.0 to 7.5; conductivity: 750 to 800 µS/cm. Zebrafish were fed 3 to 5 times a day, depending on age, with granular and live food (*Artemia salina*). Health monitoring was performed at least once a year. All embryos and larvae used in this study were raised at 28 °C and staged at 75% epiboly for synchronization. All procedures performed on animals conform to the guidelines from Directive 2010/63/EU of the European Parliament on the protection of animals used for scientific purposes and were approved by the Animal Protection Committee (Tierschutzkommission) of the Regierungspräsidium Darmstadt (reference: B2/1218).

### Statistics and Reproducibility.

Data were processed with the GraphPad Prism 9 and Microsoft Excel 2016 software. Two-sided Student’s *t* test was used to calculate *P*-values. Experiments were performed at least three times independently and displayed when showing consistent results.

### Detailed Methods Description.

A detailed description of the methods used to in this work is available in *SI Appendix*.

## Supplementary Material

Appendix 01 (PDF)

## Data Availability

Next-generation sequencing data have been deposited in the Gene Expression Omnibus (GEO) repository GSE232835 (https://www.ncbi.nlm.nih.gov/geo/query/acc.cgi?acc=GSE232835) (71). The data can be viewed at https://bioinformatics-public.mpi-bn.mpg.de/juan-et-al-2023/cellxgene-fix/ (72). All study data are included in the article and/or *SI Appendix*.

## References

[1] K. Courchaine, G. Rykiel, S. Rugonyi, Influence of blood flow on cardiac development. Prog. Biophys. Mol. Biol. **137**, 95–110 (2018). 29772208 10.1016/j.pbiomolbio.2018.05.005 PMC6109420

[2] F. Boselli, J. B. Freund, J. Vermot, Blood flow mechanics in cardiovascular development. Cell Mol. Life Sci. **72**, 2545–2559 (2015). 25801176 10.1007/s00018-015-1885-3 PMC4457920

[3] J. C. Culver, M. E. Dickinson, The effects of hemodynamic force on embryonic development. Microcirculation **17**, 164–178 (2010). 20374481 10.1111/j.1549-8719.2010.00025.x PMC2927969

[4] M. M. Collins, D. Y. Stainier, Organ function as a modulator of organ formation: Lessons from zebrafish. Curr. Top. Dev. Biol. **117**, 417–433 (2016). 26969993 10.1016/bs.ctdb.2015.10.017

[5] S. Narumanchi , Zebrafish heart failure models. Front. Cell Dev. Biol. **9**, 662583 (2021). 34095129 10.3389/fcell.2021.662583 PMC8173159

[6] R. Breckenridge, Heart failure and mouse models. Dis. Model Mech. **3**, 138–143 (2010). 20212081 10.1242/dmm.005017

[7] A. B. Arrenberg, D. Y. Stainier, H. Baier, J. Huisken, Optogenetic control of cardiac function. Science **330**, 971–974 (2010). 21071670 10.1126/science.1195929

[8] L. K. Keyt , Thin filament cardiomyopathies: A review of genetics, disease mechanisms, and emerging therapeutics. Front. Cardiovas. Med. **9**, 972301 (2022). 10.3389/fcvm.2022.972301 PMC9489950 36158814

[9] A. J. Sehnert , Cardiac troponin T is essential in sarcomere assembly and cardiac contractility. Nat. Genet. **31**, 106–110 (2002). 11967535 10.1038/ng875

[10] S. J. Rasouli, D. Y. R. Stainier, Regulation of cardiomyocyte behavior in zebrafish trabeculation by Neuregulin 2a signaling. Nat. Commun. **8**, 15281 (2017). 28485381 10.1038/ncomms15281 PMC5477525

[11] L. A. Samsa , Cardiac contraction activates endocardial Notch signaling to modulate chamber maturation in zebrafish. Development **142**, 4080–4091 (2015). 26628092 10.1242/dev.125724 PMC4712836

[12] T. Bartman , Early myocardial function affects endocardial cushion development in zebrafish. PLoS Biol. **2**, E129 (2004). 15138499 10.1371/journal.pbio.0020129 PMC406391

[13] L. Liu , Combinatorial genetic replenishments in myocardial and outflow tract tissues restore heart function in tnnt2 mutant zebrafish. Biol. Open **8**, bio046474 (2019). 31796423 10.1242/bio.046474 PMC6918781

[14] T. Natsume, M. T. Kanemaki, Conditional degrons for controlling protein expression at the protein level. Annu. Rev. Genet. **51**, 83–102 (2017). 29178817 10.1146/annurev-genet-120116-024656

[15] B. E. Housden , Loss-of-function genetic tools for animal models: Cross-species and cross-platform differences. Nat. Rev. Genet. **18**, 24–40 (2017). 27795562 10.1038/nrg.2016.118 PMC5206767

[16] G. Jakutis, D. Y. R. Stainier, Genotype-phenotype relationships in the context of transcriptional adaptation and genetic robustness. Annu. Rev. Genet. **55**, 71–91 (2021). 34314597 10.1146/annurev-genet-071719-020342

[17] M. Toure, C. M. Crews, Small-molecule PROTACS: New approaches to protein degradation. Angew. Chem. Int. Ed. Engl. **55**, 1966–1973 (2016). 26756721 10.1002/anie.201507978

[18] N. Yamaguchi, T. Colak-Champollion, H. Knaut, zGrad is a nanobody-based degron system that inactivates proteins in zebrafish. eLife **8**, e43125 (2019). 30735119 10.7554/eLife.43125 PMC6384026

[19] E. Caussinus, O. Kanca, M. Affolter, Fluorescent fusion protein knockout mediated by anti-GFP nanobody. Nat. Struct. Mol. Biol. **19**, 117–121 (2011). 22157958 10.1038/nsmb.2180

[20] M. Jussila, C. W. Boswell, N. W. Griffiths, P. G. Pumputis, B. Ciruna, Live imaging and conditional disruption of native PCP activity using endogenously tagged zebrafish sfGFP-Vangl2. Nat. Commun. **13**, 5598 (2022). 36151137 10.1038/s41467-022-33322-9 PMC9508082

[21] L. Macdonald , Rapid and specific degradation of endogenous proteins in mouse models using auxin-inducible degrons. eLife **11**, e77987 (2022). 35736539 10.7554/eLife.77987 PMC9273210

[22] K. J. Clark , In vivo protein trapping produces a functional expression codex of the vertebrate proteome. Nat. Methods **8**, 506–515 (2011). 21552255 10.1038/nmeth.1606 PMC3306164

[23] L. Burg , Conditional mutagenesis by oligonucleotide-mediated integration of loxP sites in zebrafish. PLoS Genet. **14**, e1007754 (2018). 30427827 10.1371/journal.pgen.1007754 PMC6261631

[24] A. Nickless, J. M. Bailis, Z. You, Control of gene expression through the nonsense-mediated RNA decay pathway. Cell Biosci. **7**, 26 (2017). 28533900 10.1186/s13578-017-0153-7 PMC5437625

[25] F. Liu , Cre/lox regulated conditional rescue and inactivation with zebrafish UFlip alleles generated by CRISPR-Cas9 targeted integration. eLife **11**, e71478 (2022). 35713402 10.7554/eLife.71478 PMC9270027

[26] K. Sugimoto, S. P. Hui, D. Z. Sheng, K. Kikuchi, Dissection of zebrafish shha function using site-specific targeting with a Cre-dependent genetic switch. eLife **6**, e24635 (2017). 28513431 10.7554/eLife.24635 PMC5435461

[27] J. Li , One-step generation of zebrafish carrying a conditional knockout-knockin visible switch via CRISPR/Cas9-mediated intron targeting. Sci. China Life Sci. **63**, 59–67 (2020). 31872378 10.1007/s11427-019-1607-9

[28] W. Li , One-step efficient generation of dual-function conditional knockout and geno-tagging alleles in zebrafish. eLife **8**, e48081 (2019). 31663848 10.7554/eLife.48081 PMC6845224

[29] M. Shin , Generation and application of endogenously floxed alleles for cell-specific knockout in zebrafish. Dev. Cell **58**, 2614–2626.e7 (2023), 10.1016/j.devcel.2023.07.022. 37633272 PMC10840978

[30] K. Kikuchi , Primary contribution to zebrafish heart regeneration by gata4(+) cardiomyocytes. Nature **464**, 601–605 (2010). 20336144 10.1038/nature08804 PMC3040215

[31] P. Han , Coordinating cardiomyocyte interactions to direct ventricular chamber morphogenesis. Nature **534**, 700–704 (2016). 27357797 10.1038/nature18310 PMC5330678

[32] R. Zhang, X. Xu, Transient and transgenic analysis of the zebrafish ventricular myosin heavy chain (vmhc) promoter: An inhibitory mechanism of ventricle-specific gene expression. Dev. Dyn. **238**, 1564–1573 (2009). 19322764 10.1002/dvdy.21929 PMC2756512

[33] R. Zhang , In vivo cardiac reprogramming contributes to zebrafish heart regeneration. Nature **498**, 497–501 (2013). 23783515 10.1038/nature12322 PMC4090927

[34] C. D. Hsiao, W. Y. Tsai, L. S. Horng, H. J. Tsai, Molecular structure and developmental expression of three muscle-type troponin T genes in zebrafish. Dev. Dyn. **227**, 266–279 (2003). 12761854 10.1002/dvdy.10305

[35] J. Li , Intron targeting-mediated and endogenous gene integrity-maintaining knockin in zebrafish using the CRISPR/Cas9 system. Cell Res. **25**, 634–637 (2015). 25849248 10.1038/cr.2015.43 PMC4423083

[36] J. M. Welker , GeneWeld: Efficient targeted integration directed by short homology in zebrafish. Bio. Protoc. **11**, e4100 (2021). 10.21769/BioProtoc.4100 PMC8329467 34395736

[37] M. Lange , Zebrahub–Multimodal zebrafish developmental atlas reveals the state transition dynamics of late vertebrate pluripotent axial progenitors. bioXriv [Preprint] (2023). 10.1101/2023.03.06.531398 (Accessed 15 June 2023). 39454574

[38] C. J. Huang, C. T. Tu, C. D. Hsiao, F. J. Hsieh, H. J. Tsai, Germ-line transmission of a myocardium-specific GFP transgene reveals critical regulatory elements in the cardiac myosin light chain 2 promoter of zebrafish. Dev. Dyn. **228**, 30–40 (2003). 12950077 10.1002/dvdy.10356

[39] M. Peralta , Heartbeat-driven pericardiac fluid forces contribute to epicardium morphogenesis. Curr. Biol. **23**, 1726–1735 (2013). 23954432 10.1016/j.cub.2013.07.005

[40] J. Vermot , Reversing blood flows act through klf2a to ensure normal valvulogenesis in the developing heart. PLoS Biol. **7**, e1000246 (2009). 19924233 10.1371/journal.pbio.1000246 PMC2773122

[41] J. Gerdes , Cell cycle analysis of a cell proliferation-associated human nuclear antigen defined by the monoclonal antibody Ki-67. J. Immunol. **133**, 1710–1715 (1984). 6206131

[42] F. C. Serluca, Development of the proepicardial organ in the zebrafish. Dev. Biol. **315**, 18–27 (2008). 18206866 10.1016/j.ydbio.2007.10.007

[43] D. S. Peal, C. G. Burns, C. A. Macrae, D. Milan, Chondroitin sulfate expression is required for cardiac atrioventricular canal formation. Dev. Dyn. **238**, 3103–3110 (2009). 19890913 10.1002/dvdy.22154 PMC2852642

[44] F. Fontana , Antagonistic activities of Vegfr3/Flt4 and Notch1b fine-tune mechanosensitive signaling during zebrafish cardiac valvulogenesis. Cell Rep. **32**, 107883 (2020). 32668254 10.1016/j.celrep.2020.107883

[45] Y. T. Lee, L. He, Y. Zhou, Expanding the chemogenetic toolbox by circular permutation. J. Mol. Biol. **432**, 3127–3136 (2020). 32277990 10.1016/j.jmb.2020.03.033 PMC7710497

[46] Z. Liu , Systematic comparison of 2A peptides for cloning multi-genes in a polycistronic vector. Sci. Rep. **7**, 2193 (2017). 28526819 10.1038/s41598-017-02460-2 PMC5438344

[47] K. Mattonet , Endothelial versus pronephron fate decision is modulated by the transcription factors Cloche/Npas4l, Tal1, and Lmo2. Sci. Adv. **8**, eabn2082 (2022). 36044573 10.1126/sciadv.abn2082 PMC9432843

[48] A. Guerra , Distinct myocardial lineages break atrial symmetry during cardiogenesis in zebrafish. eLife **7**, e32833 (2018). 29762122 10.7554/eLife.32833 PMC5953537

[49] F. Tessadori , Twisting of the zebrafish heart tube during cardiac looping is a tbx5-dependent and tissue-intrinsic process. eLife **10**, e61733 (2021). 34372968 10.7554/eLife.61733 PMC8354640

[50] A. L. Duchemin, H. Vignes, J. Vermot, Mechanically activated piezo channels modulate outflow tract valve development through the Yap1 and Klf2-Notch signaling axis. eLife **8**, e44706 (2019). 31524599 10.7554/eLife.44706 PMC6779468

[51] A. C. Dietrich, V. A. Lombardo, J. Veerkamp, F. Priller, S. Abdelilah-Seyfried, Blood flow and Bmp signaling control endocardial chamber morphogenesis. Dev. Cell **30**, 367–377 (2014). 25158852 10.1016/j.devcel.2014.06.020

[52] E. de Pater , Distinct phases of cardiomyocyte differentiation regulate growth of the zebrafish heart. Development **136**, 1633–1641 (2009). 19395641 10.1242/dev.030924 PMC2673760

[53] N. Yucel , Cardiac endothelial cells maintain open chromatin and expression of cardiomyocyte myofibrillar genes. eLife **9**, e55730 (2020). 33315013 10.7554/eLife.55730 PMC7758065

[54] E. Steed, F. Boselli, J. Vermot, Hemodynamics driven cardiac valve morphogenesis. Biochim. Biophys. Acta **1863**, 1760–1766 (2016). 26608609 10.1016/j.bbamcr.2015.11.014

[55] T. Haack, S. Abdelilah-Seyfried, The force within: Endocardial development, mechanotransduction and signalling during cardiac morphogenesis. Development **143**, 373–386 (2016). 26839341 10.1242/dev.131425

[56] K. Nishimura, T. Fukagawa, H. Takisawa, T. Kakimoto, M. Kanemaki, An auxin-based degron system for the rapid depletion of proteins in nonplant cells. Nat. Methods **6**, 917–922 (2009). 19915560 10.1038/nmeth.1401

[57] L. Zhang, J. D. Ward, Z. Cheng, A. F. Dernburg, The auxin-inducible degradation (AID) system enables versatile conditional protein depletion in C. elegans. Development **142**, 4374–4384 (2015). 26552885 10.1242/dev.129635 PMC4689222

[58] A. Yesbolatova , The auxin-inducible degron 2 technology provides sharp degradation control in yeast, mammalian cells, and mice. Nat. Commun. **11**, 5701 (2020). 33177522 10.1038/s41467-020-19532-z PMC7659001

[59] B. Gu, E. Posfai, J. Rossant, Efficient generation of targeted large insertions by microinjection into two-cell-stage mouse embryos. Nat. Biotechnol. **36**, 632–637 (2018). 29889212 10.1038/nbt.4166

[60] M. Trost, A. C. Blattner, C. F. Lehner, Regulated protein depletion by the auxin-inducible degradation system in Drosophila melanogaster. Fly **10**, 35–46 (2016). 27010248 10.1080/19336934.2016.1168552 PMC4934730

[61] K. Daniel , Conditional control of fluorescent protein degradation by an auxin-dependent nanobody. Nat. Commun. **9**, 3297 (2018). 30120238 10.1038/s41467-018-05855-5 PMC6098157

[62] N. Jullien, F. Sampieri, A. Enjalbert, J. P. Herman, Regulation of Cre recombinase by ligand-induced complementation of inactive fragments. Nucleic Acids Res. **31**, e131 (2003). 14576331 10.1093/nar/gng131 PMC275488

[63] B. Zetsche, S. E. Volz, F. Zhang, A split-Cas9 architecture for inducible genome editing and transcription modulation. Nat. Biotechnol. **33**, 139–142 (2015). 25643054 10.1038/nbt.3149 PMC4503468

[64] H. Götzke , The ALFA-tag is a highly versatile tool for nanobody-based bioscience applications. Nat. Commun. **10**, 4403 (2019). 31562305 10.1038/s41467-019-12301-7 PMC6764986

[65] N. Zhao , A genetically encoded probe for imaging nascent and mature HA-tagged proteins in vivo. Nat. Commun. **10**, 2947 (2019). 31270320 10.1038/s41467-019-10846-1 PMC6610143

[66] M. E. Tanenbaum, L. A. Gilbert, L. S. Qi, J. S. Weissman, R. D. Vale, A protein-tagging system for signal amplification in gene expression and fluorescence imaging. Cell **159**, 635–646 (2014). 25307933 10.1016/j.cell.2014.09.039 PMC4252608

[67] K. M. Sathyan , An improved auxin-inducible degron system preserves native protein levels and enables rapid and specific protein depletion. Genes Dev. **33**, 1441–1455 (2019). 31467088 10.1101/gad.328237.119 PMC6771385

[68] J. E. Wilcox, R. E. Hershberger, Genetic cardiomyopathies. Curr. Opin. Cardiol. **33**, 354–362 (2018). 29561320 10.1097/HCO.0000000000000512

[69] R. Yotti, C. E. Seidman, J. G. Seidman, Advances in the genetic basis and pathogenesis of Sarcomere Cardiomyopathies. Annu. Rev. Genom. Hum. Genet. **20**, 129–153 (2019). 10.1146/annurev-genom-083118-015306 30978303

[70] B. Gangadharan , Molecular mechanisms and structural features of cardiomyopathy-causing troponin T mutants in the tropomyosin overlap region. Proc. Natl. Acad. Sci. U.S.A. **114**, 11115–11120 (2017). 28973951 10.1073/pnas.1710354114 PMC5651771

[71] T. Juan , Gene expression profile at single cell level of 72 hpf wild-type, tnnt2a mutant, and Tnnt2a degron zebrafish larvae. Gene Expression Omnibus Repository. https://www.ncbi.nlm.nih.gov/geo/query/acc.cgi?acc=GSE232835. Deposited 18 May 2023.

[72] T. Juan , Gene expression profile at single cell level of 72 hpf wild-type, tnnt2a mutant, and Tnnt2a degron zebrafish larvae. CELLXGENE Visualization Tool. https://bioinformatics-public.mpi-bn.mpg.de/juan-et-al-2023/cellxgene-fix/. Deposited 22 May 2023.

