# Supplementary material for: Control of cardiac contractions using Cre-lox and degron strategies in zebrafish
Source: Proc Natl Acad Sci U S A. 2024 Jan 9;121(3):e2309842121. doi: 10.1073/pnas.2309842121 (PMC10801847; doi:10.1073/pnas.2309842121)
Supplement: Supplementary file 1 — Appendix 01 (PDF) [file pnas.2309842121.sapp.pdf]

Supplementary information appendix

SI Legends

Fig. S1 Floxed *tnnt2a* recombination leads to a 9 kb deletion. (A) Expected and sequenced CRISPR/Cas9 insertion of the second LOXP site in the recombined *tnnt2a^{mn0031Gt}* locus; arrowheads point to Cas9 cleavage site. (B) Agarose gel image and schematic of the *tnnt2a* locus showing a PCR amplification of the *tnnt2a* floxed region from 48 hpf wild types and *tnnt2a$^{flox/flox}$* embryos non-injected or injected at the one-cell stage with *Cre* mRNA. (C) Agarose gel image and schematic of *tnnt2a* mRNA showing a PCR amplification of *tnnt2a* full-length mRNA from 48 hpf wild types and *tnnt2a$^{flox/flox}$* embryos non-injected or injected at the one-cell stage with *Cre* mRNA. (D) Relative *tnnt2a* mRNA levels in 48 hpf wild types and *tnnt2a$^{flox/flox}$* embryos non-injected or injected at the one-cell stage with *Cre* mRNA; n= 3 biologically independent samples; Ct values are listed in Supplementary file 1; error bars indicate s.d. and a two-sided Student's t-test was used to calculate the p-values. Arrowhead on *tnnt2a* locus and mRNA diagrams indicate the primer binding sites, with the expected amplicon size indicated below the reverse primer. Grey "Stop" is a PTC.

Fig. S2 Myocardial Cre-lox-mediated *tnnt2a* deletion occurs too late to produce early cardiac contraction defects. (A) Brightfield image of a 120 hpf *tnnt2a$^{flox/flox}$* ; *myl7:Cre$^{+/-}$* larva; asterisk indicates the absence of pericardial edema. (A') Brightfield image and kymograph of a heart from a 120 hpf *tnnt2a$^{flox/flox}$*; *myl7:Cre$^{+/-}$* larva; green lines outline the ventricle (V), blue lines outline the atrium (A), and vertical white lines indicate the reference axis of the kymographs. (B-B'), Brightfield images of 24, 48, 72, and 120 hpf *tnnt2a$^{flox/flox}$* (B) and *tnnt2a$^{flox/flox}$* ; *myl7:Cre$^{+/-}$* (B') embryos and larvae, stained for *tnnt2a* expression; asterisk and arrowhead indicate respectively wild-type and decreased *tnnt2a* mRNA levels in the heart. (C) Agarose gel image showing a PCR amplification of the *tnnt2a* floxed region from 24 and 48 hpf *tnnt2a$^{flox/flox}$*; *myl7:Cre$^{+/-}$* embryos. (D-G) Confocal images of hearts from 24 and 48 hpf *myl7:Cre$^{+/-}$* (D), *myl7:Cre-ERT2$^{+/-}$* (E), *myh7:zfCre-ERT2$^{+/-}$* (F), and *myh6:Cre-ERT2$^{+/-}$* (G) embryos; all are also *ubb:LOXP-eGFP-LOXP-mCherry$^{+/-}$* ; *Cre-ERT2*+ embryos were treated with 4-OHT; maximum projection of a 40 µm z-section; annotations correspond to the ventricle (V) and atrium (A). 'Red hot' lookup table coloring (from Low to High) highlights the standard deviation (A) or 3D variance (A'). Diagrams indicate the Anterior-Posterior (A-P), Dorsal-Ventral (D-V), and Left-Right (L-R) axes.

Fig. S3 Deletion of *tnnt2a* at 6 hpf, but not at 24 hpf, induces early cardiac contraction defects. (A) Schematic of *tnnt2a^{flox}* recombination strategy in *hsp70l:Cre* background; recombination is achieved by heat shock treatments at 6 or 24 hpf. (B) Brightfield images of 24 hpf *tnnt2a$^{flox/flox}$; hsp70l:Cre$^{+/-}$* embryos, not heat shocked (No HS) or heat shocked at 6 hpf (HS) ; arrowheads and asterisks indicate respectively the presence and absence of pericardial edema. (C-E) Brightfield images of 120 hpf *tnnt2a$^{flox/flox}$; hsp70l:Cre$^{+/-}$* larvae not heat shocked (C), or heat shocked at 6 (D) or 24 (E) hpf; arrowhead and asterisks indicate respectively the presence and absence of pericardial edema. (C'-E') Brightfield images and kymographs of 120 hpf hearts from *tnnt2a$^{flox/flox}$; hsp70l:Cre$^{+/-}$* larvae, not heat shocked (C'), or heat shocked at 6 (D') or 24 (E') hpf; green lines outline the ventricle (V), blue lines outline the atrium (A), and vertical white lines indicate the reference axis of the kymographs. 'Red hot' lookup table coloring (from Low to High) highlights the standard deviation (C,D,E) or 3D variance (C',D',E'). Diagrams indicate the Anterior-Posterior (A-P), Dorsal-Ventral (D-V), and Left-Right (L-R) axes.

Fig. S4 Concatemer removal in the *bns511 tnnt2a* knock-in allele restores *tnnt2a* mRNA towards wild-type levels. (A) Expected and sequenced CRISPR/Cas9 insertion of the *eGFP*-containing cassette in *tnnt2a* last intron; arrowheads point to Cas9 cleavage site. (B) Agarose gel image and schematic of *tnnt2a* locus showing a PCR amplification of the *eGFP*-containing region on genomic DNA from 48 hpf wild-type, $tnnt2a^{bns511/bns511}$, and $tnnt2a^{bns513/bns513}$ embryos. (C) Agarose gel image and schematic of *tnnt2a* mRNA showing a PCR amplification of *tnnt2a* full-length mRNA on cDNA from 48 hpf wild-type, $tnnt2a^{bns511/bns511}$ and $tnnt2a^{bns513/bns513}$ embryos; proportions of wild-type *tnnt2a* compared with total *tnnt2a* PCR amplicons after cloning and sequencing are displayed on the agarose gel image. (D) Relative *tnnt2a* mRNA levels in 48 hpf wild-type, $tnnt2a^{bns511/bns511}$ and $tnnt2a^{bns513/bns513}$ embryos; n= 3 biologically independent samples; Ct values are listed in Supplementary file 1; error bars indicate s.d. and a two-sided Student's t-test was used to calculate the p-values. Arrows on *tnnt2a* locus and mRNA diagrams indicate the primer binding sites, with the expected amplicon size indicated below the reverse primer.

Fig. S5 Myocardial Tnnt2a-eGFP degradation can occur during the early stages of heart development. (A) Western blot detection of Tnnt2a-eGFP (61.06 kDa) in 48 hpf $tnnt2a^{bns513/bns513}$, *myl7:zGRAD-P2A-TagBFP*$^{+/-}$, $tnnt2a^{bns513/bns513}$; *myl7:zGRAD-P2A-TagBFP*$^{+/-}$, and AB wild-type embryos. (B-D) Confocal images of hearts from 36 and 24 hpf $tnnt2a^{bns513/bns513}$ (B), *myl7:zGRAD-P2A-TagBFP*$^{+/-}$ (C), and $tnnt2a^{bns513/bns513}$; *myl7:zGRAD-P2A-TagBFP*$^{+/-}$ (D) embryos; maximum z-projection; annotations correspond to the ventricle (V) and atrium (A); diagrams indicate the Anterior-Posterior (A-P) and Left-Right (L-R) axes.

Fig. S6 Similar blood flow-dependent effects in Tnnt2a degrons and *tnnt2a* mutants. (A) UMAP representation of representative markers of multiple cell populations. (B) Respective cell number of cell populations in wild-type, *tnnt2a* mutant, and Tnnt2a degron hearts at 72 hpf. (C-F) UMAP representation of data merged or split by genotype of the cell proliferation marker *mki67* (C), the epicardial marker *tcf21* (D), the endocardial valve marker *spp1* (E), and the flow-repressed endothelial marker *flt4* (F); arrowheads point to regions of expression of the displayed markers.

Fig. S7 *tnnt2a* is expressed in endocardial valve cells. (A) UMAP representation of *tnnt2a* expression in wild-type, *tnnt2a* mutant, and Tnnt2a degron hearts at 72 hpf; from left to right, arrowheads point to the myocardial, smooth muscle, and endocardial valve cell populations. (B) Confocal images of heart sections centered on the atrioventricular valve from 72 and 120 hpf $tnnt2a^{mn0031Gt/bns513}$ larvae; diagram indicates the Anterior-Posterior (A-P) and Left-Right (L-R) axes.

Fig. S8 Temporal control of *zGRAD* expression fails to induce Tnnt2a-eGFP degradation. (A-D') Confocal images of hearts from 48 hpf $tnnt2a^{bns513/bns513}$ (A), $tnnt2a^{bns513/bns513}$; *hsp70l:zGRAD-IRES-h2a-TagBFP*$^{+/-}$ heat-shocked at 36 hpf (B), $tnnt2a^{bns513/bns513}$; *myl7:Cre-ERT2*$^{+/-}$; *actb2:LOXP-mCherry-LOXP-zGRAD*$^{+/-}$ treated with DMSO (C) or 4-OHT (C'), and $tnnt2a^{bns513/bns513}$; *myl7:Cre-ERT2*$^{+/-}$; *myl7:LOXP-stop-LOXP-zGRAD-P2A-TagBFP*$^{+/-}$ treated with DMSO (D) or 4-OHT (D'); maximum z-projection. Diagram indicates the Anterior-Posterior (A-P) and Left-Right (L-R) axes.

Fig. S9 Transient myocardial expression of the split-zGRAD system enables Tnnt2a-eGFP degradation. (A-C) Confocal images of hearts from $tnnt2a^{bns513/bns513}$ animals injected at the one-cell stage with a plasmid encoding a *myl7:split-zGRAD* construct (A), a *myh7:split-zGRAD* construct (B), and a *myh6:split-zGRAD* construct (C) and treated with rapamycin

from 48 to 72 hpf; white lines highlight the TagBFP-positive cells in the eGFP channel. Diagram indicates the Anterior-Posterior (A-P) and Left-Right (L-R) axes.

Supplementary Table S1. CRISPR sites, primers, donors, and Ct values.

Fig. S1 Floxed *tnnt2a* recombination leads to a 9 kb deletion.

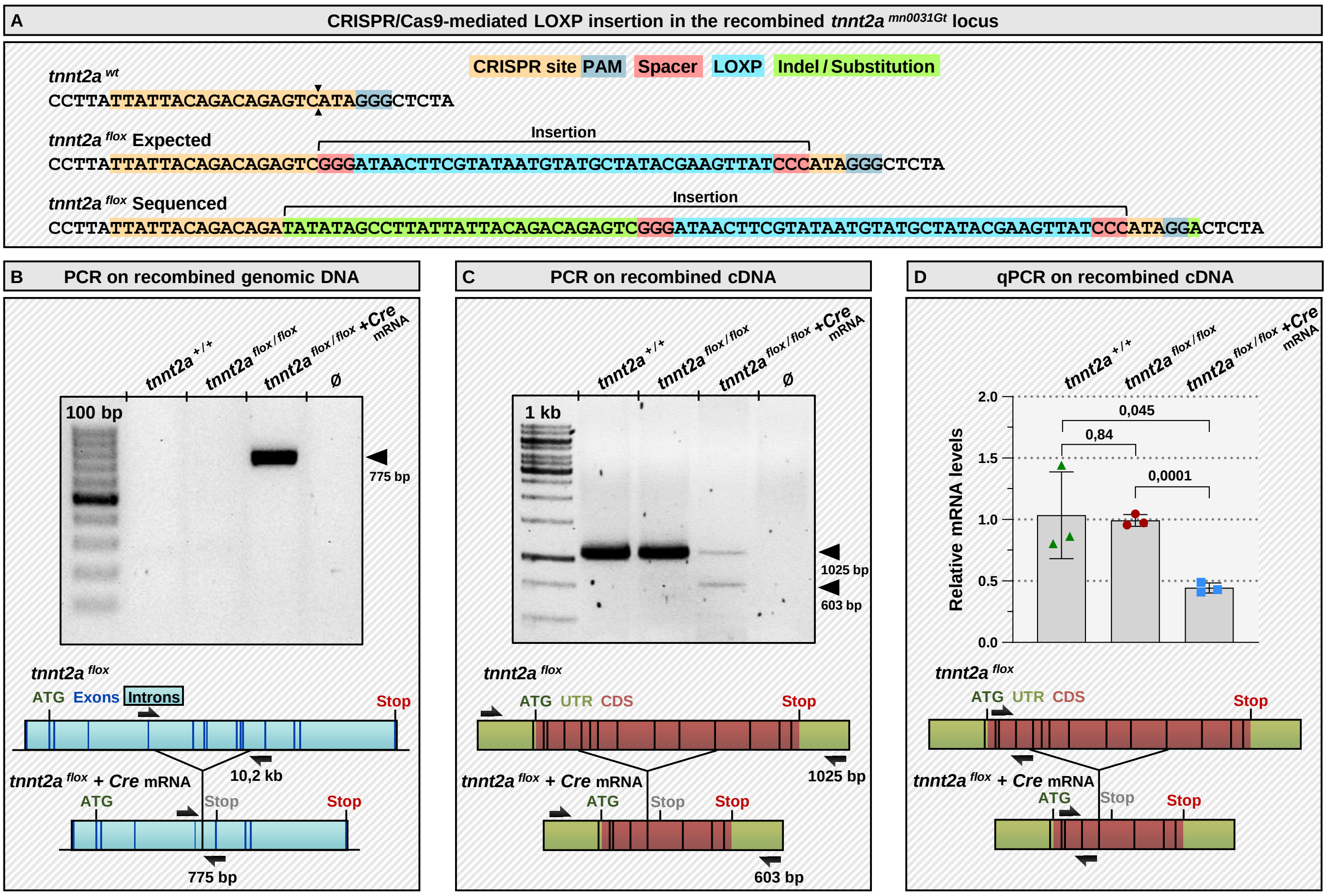

Fig. S2 Myocardial Cre-lox-mediated *tnnt2a* deletion occurs too late to produce early cardiac contraction defects.

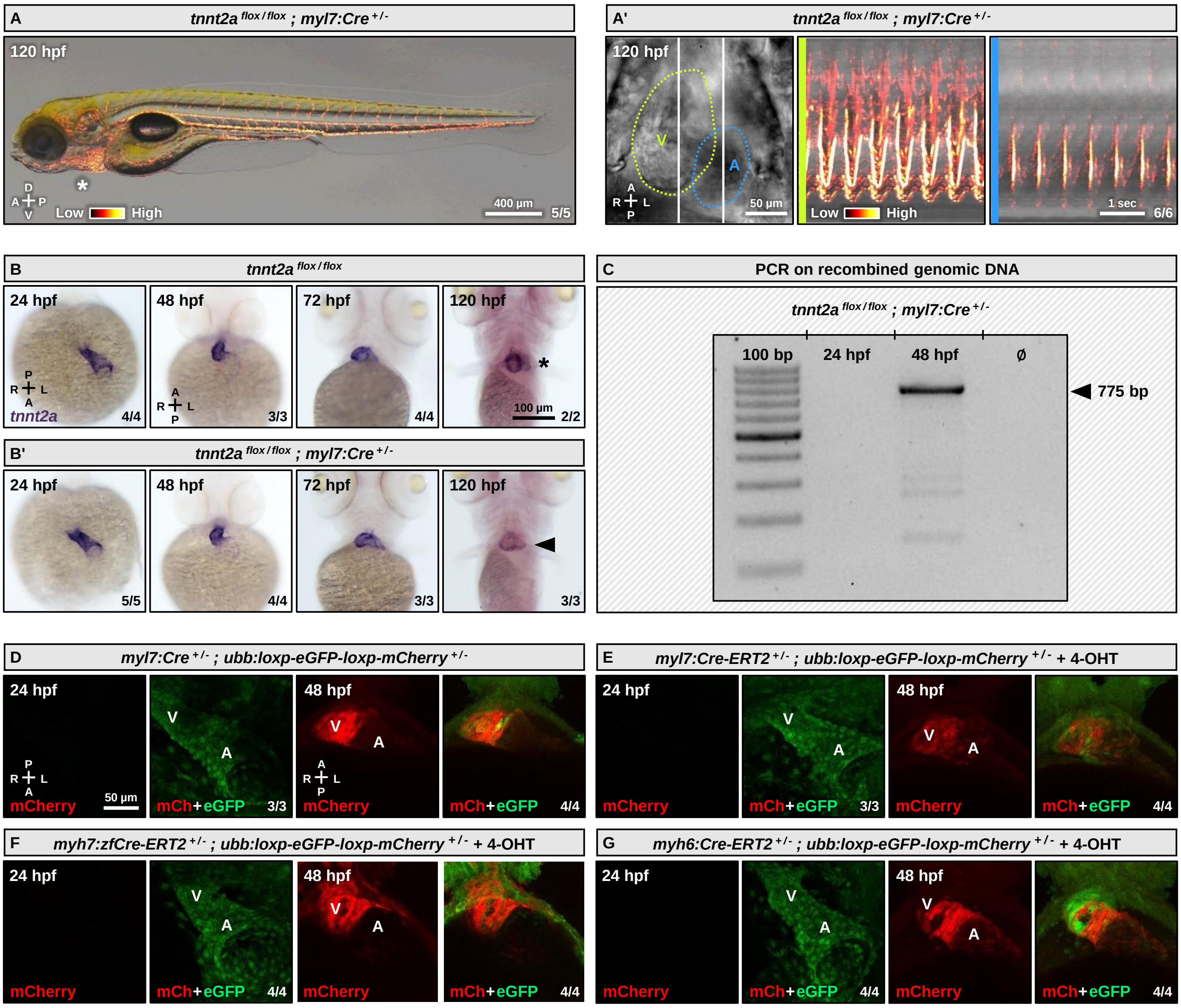

Fig. S3 Deletion of *tnnt2a* at 6 hpf, but not 24 hpf, induces early cardiac contraction defects.

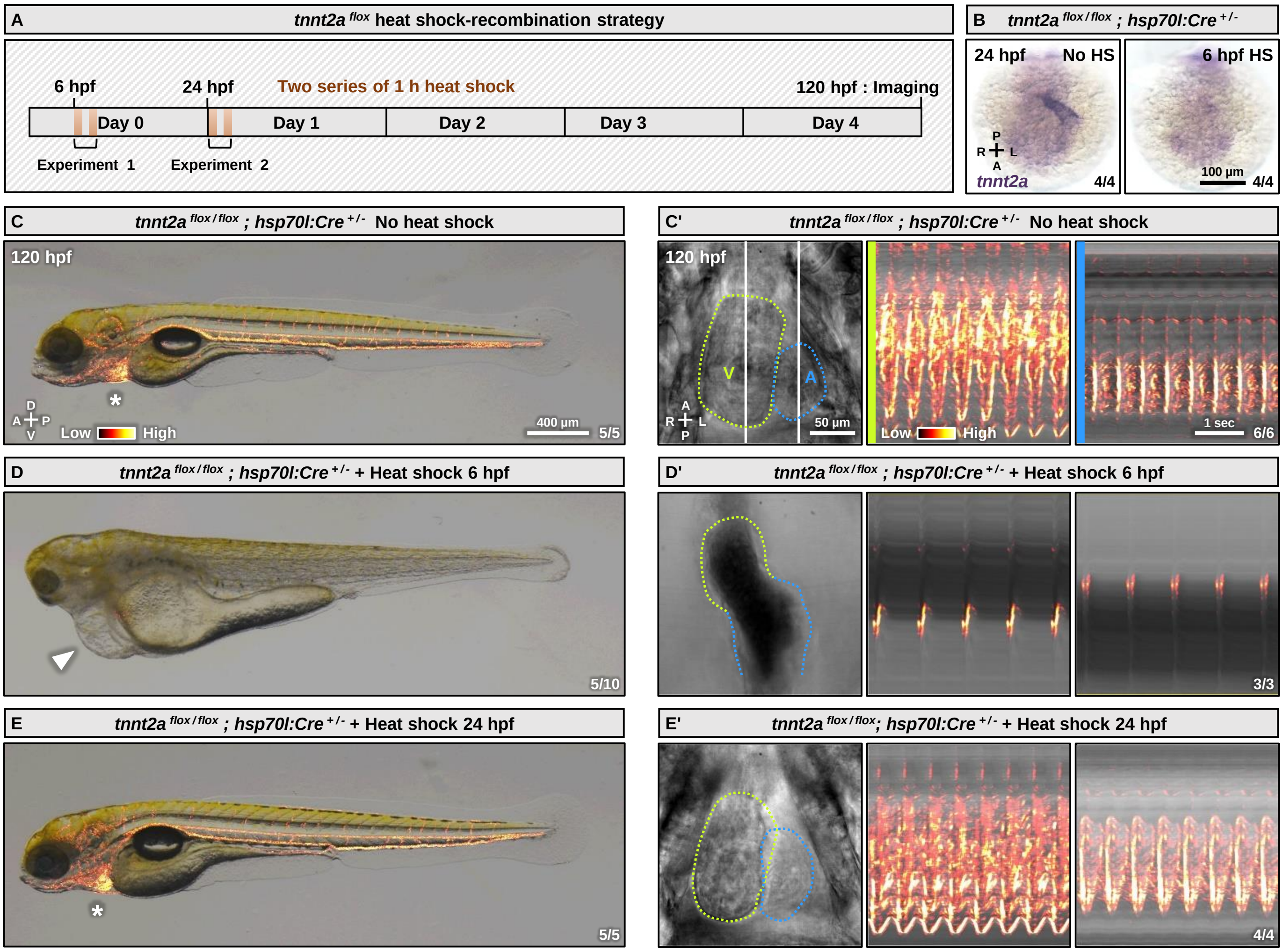

Fig. S4 Concatemer removal in the *bns511 tnnt2a* knock-in allele restores *tnnt2a* mRNA towards wild-type levels.

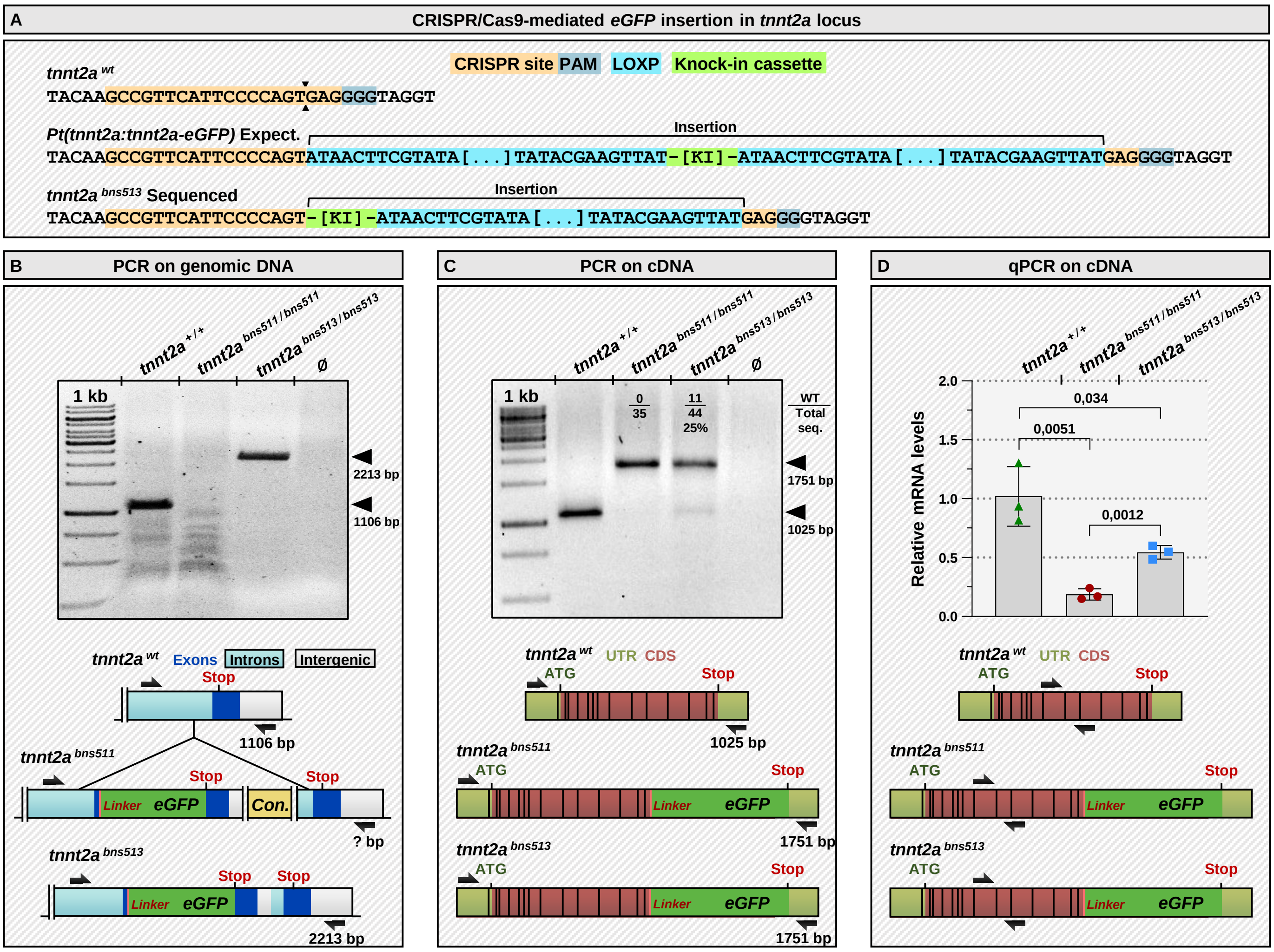

Fig. S5 Myocardial Tnnt2a-eGFP degradation can occur during the early stages of heart development.

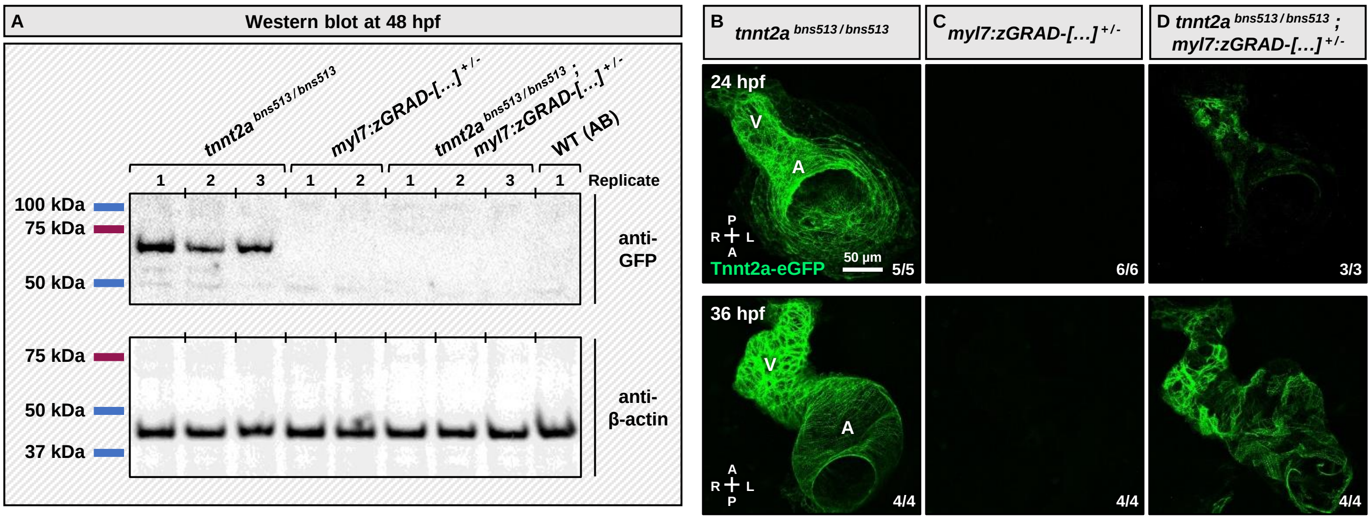

Fig. S6 Similar blood-flow dependent effects in the Tnnt2a degrons and *tnnt2a* mutants.

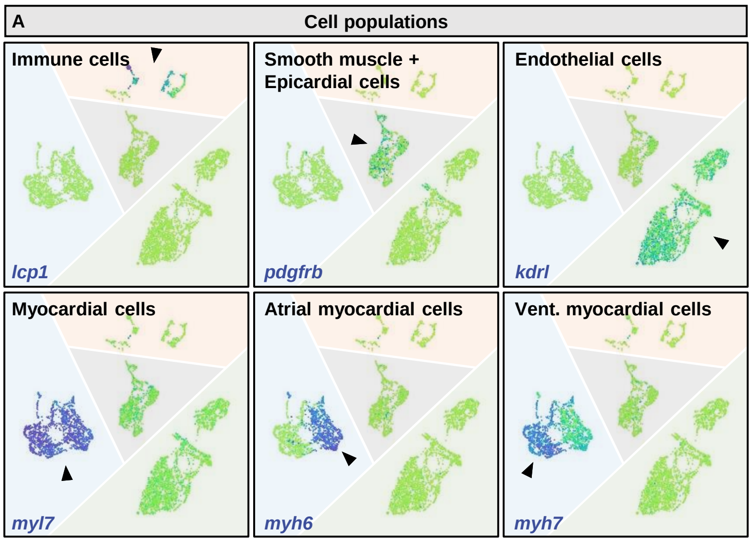

B Cell number

Cell types		WT	Mutant	Degron
Smooth muscle cells + Epicardial cells	Proliferating	119	5	4
	Smooth muscle	315	69	205
	Epicardial	179	7	6
Endothelial cells	Proliferating	483	18	18
	Valve	148	0	15
	Others	774	363	439
Myocardial cells	Proliferating	49	7	8
	Atrial	194	345	211
	Ventricular	155	312	223

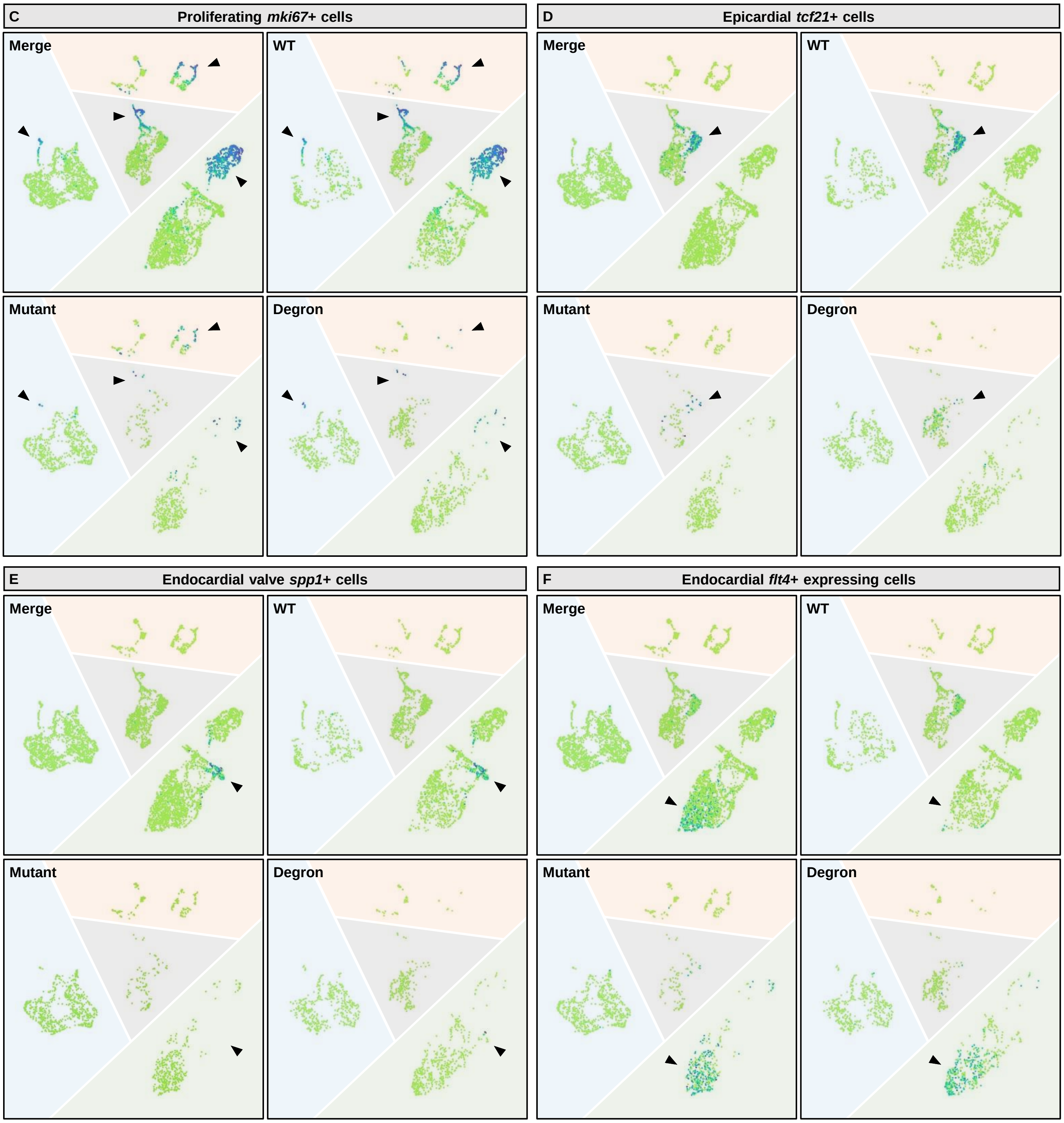

Fig. S7 *tnnt2a* is expressed in endocardial valve cells

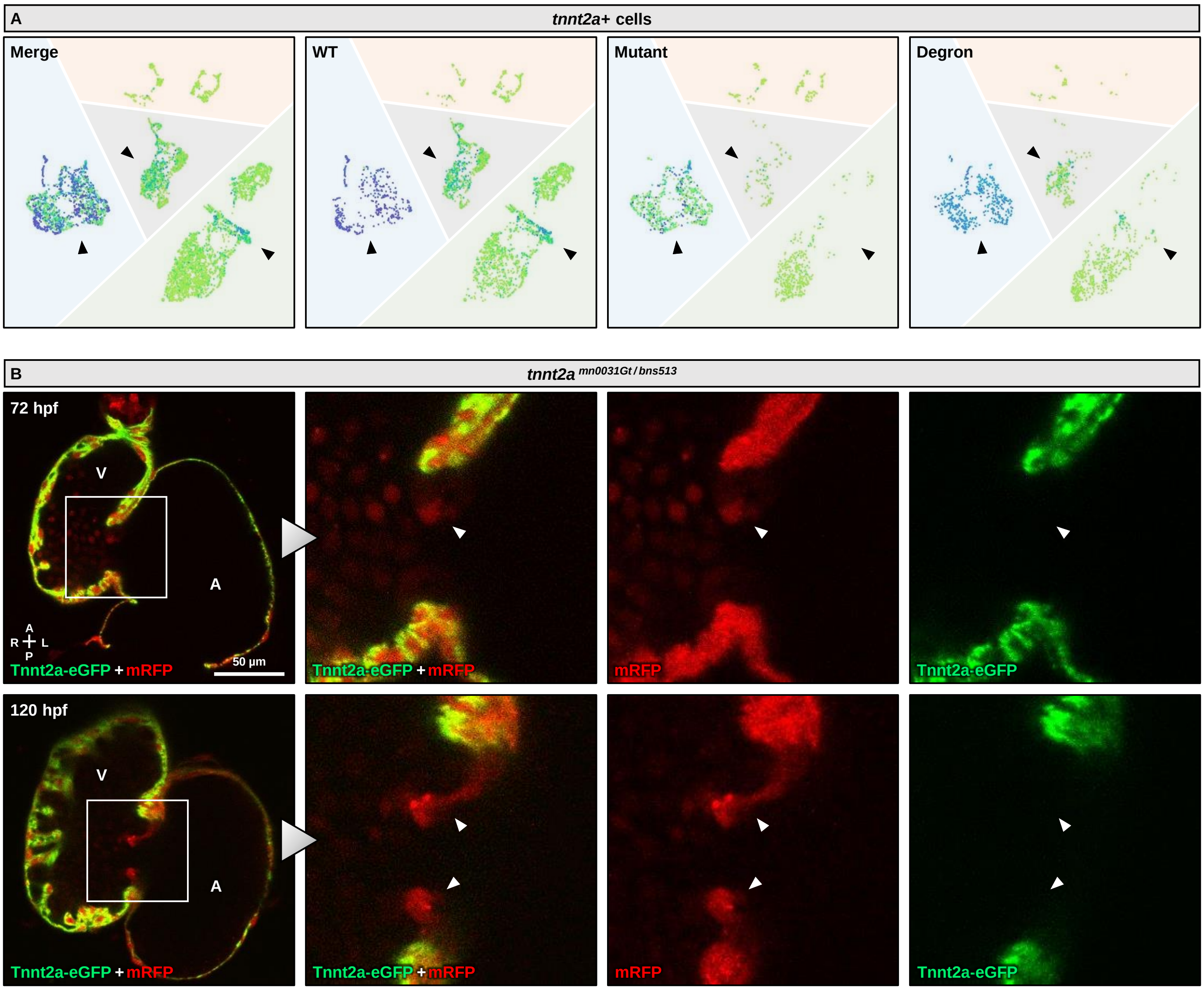

Fig. S8 Temporal control of *zGRAD* expression fails to induce Tnnt2a-eGFP degradation.

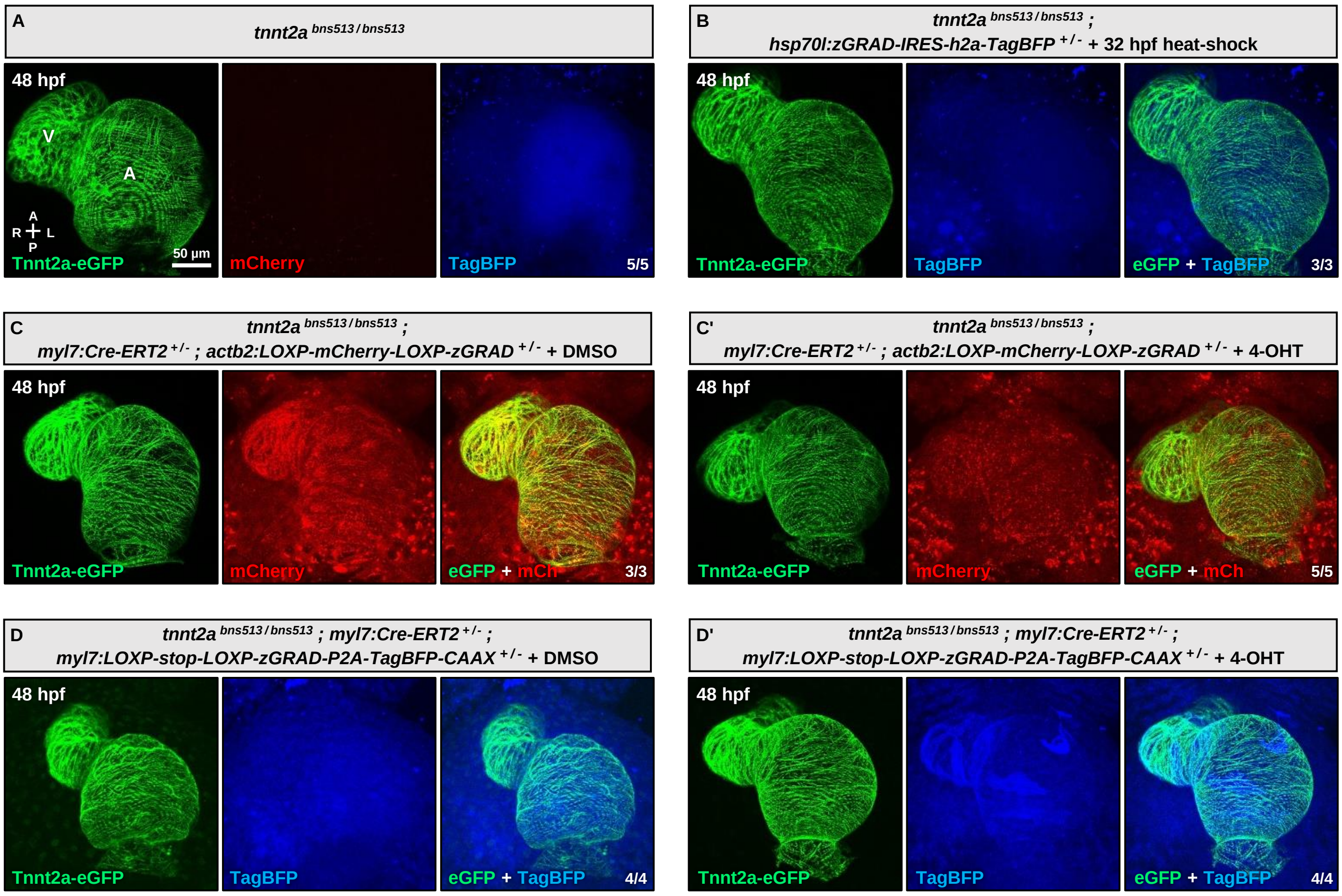

Fig. S9 Transient myocardial expression of the split-zGRAD system enables Tnnt2a-eGFP degradation.

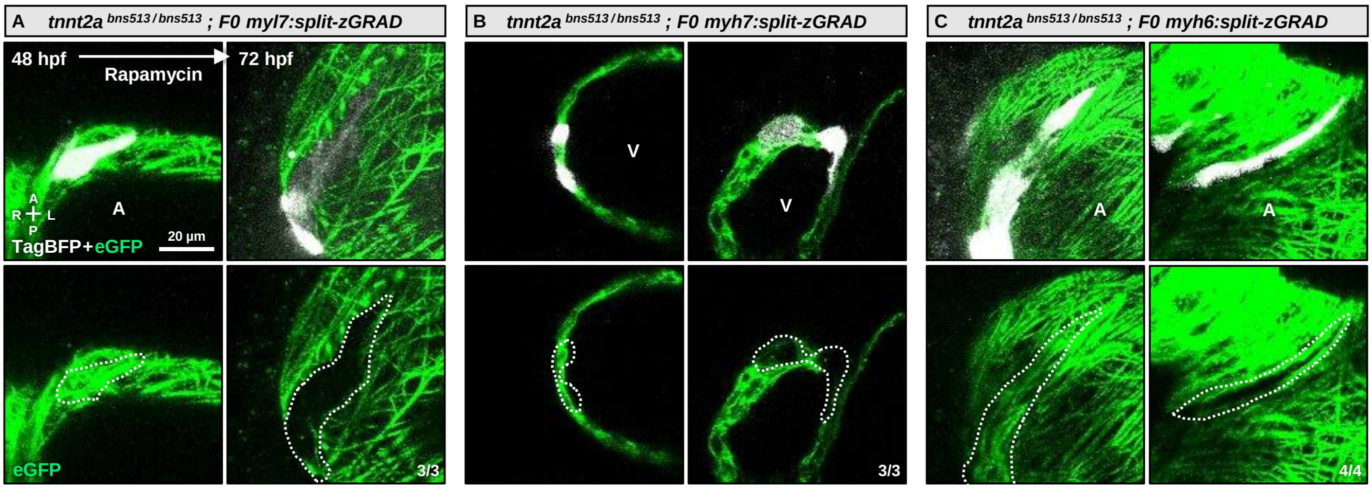

Supplementary Table S1. CRISPR sites, primers, donors, and Ct values.

Crispr/Cas9 target	CRISPR site with PAM (5'-3')	Flanking primer 1 (5'-3')	Flanking primer 2 (5'-3')
Knock-in of LOXP site in the intron 11 of tnnt2a	TTATTACAGACAGAGTCATAGGG	AGAGAGCAGAAGATGATGCCAAGAAG	GGTTGTCCAGAGTAATTGTGATGTCC
Knock-in of eGFP in the last intron of tnnt2a	GCCGTTCATTCCCCAGTGAGGGG	CTGAGTCACTGCAAAGATGTATGG	CAGCCATTGTTTGAGCTCATTTGC

Knock-in target	Donor ssODN (5'-3') Homology arms/**Spacer**
Knock-in of LOXP site in the intron 11 of tnnt2a	TACAGACAGAGTC**GGG**ATAACTTCGTATAATGTATG CTATACGAAGTTAT**CCC**ATAGGGCTCTATTTTGACG GT

PCR	Primer 1 (5'-3')	Primer 2 (5'-3')
tnnt2a full length cDNA (Fig. S1C,S4C)	CTGAGCTGGTTTTCCTCTGCATC	CAGGTAAAATCTACATAGTTCAGTG
tnnt2a^{tc300b} mutant specific	ATGTCAGACAACGAAGAAGTGGAAG	CATAAGACTTACCCTCCTGCTCCCC
tnnt2a^{floxed} wild-type specific (*mn0031Gt* flanking)	GTTGCCATGTTTGAAAGCTGTGTGC	TCCATGAAAAAACAGAAGCCGTCAGT
tnnt2a^{floxed} transgene specific (*mn0031Gt* specific)	GTTGCCATGTTTGAAAGCTGTGTGC	GACTAATACACCTCTTCCCGCATCG
tnnt2a^{floxed} recombination specific (P1 on *mn0031Gt)* (Fig. S1B,S2C)	CGATGCGGGAAGAGGTGTATTAGTC	GGTTGTCCAGAGTAATTGTGATGTCC
tnnt2a^{bns513} wild-type specific (Fig. S4B)	CTGAGTCACTGCAAAGATGTATGG	CAGCCATTGTTTGAGCTCATTTGC
tnnt2a^{bns513} transgene specific	CTGAGTCACTGCAAAGATGTATGG	GCTGAACTTGTGGCCGTTTAC
Cre specific	CCAATTTACTGACCGTACACC	ATCTTCCAGCAGGCGCACCATTG
zGrad specific	GAGACGGAGATGGAGGACAAAAC	GCTGGAGACGGTGACCTGGGTG

In situ hybridization	Primer 1 (5'-3') T3 promoter	Primer 2 (5'-3') T7 promoter
tnnt2a probe	cgcaattaaccctcactaaagggATGGAGAAGAGGA GACAGAAGATGG	cgctaatacgactcactatagggCTTTTGCATGTAA CCGCCGAAG

RT-qPCR target	Primer 1 (5'-3')	Primer 2 (5'-3')
eef1b2	ATCTGTTTGGCTCCGATGAG	CAGGCTTCTTTGCCTTCTTG
tnnt2a (Fig. S1D)	AAGTGGAAGAGTACGAGGAGCAG	GTTCCTGAGTGGTCTCTTCATCTC
tnnt2a (Fig. S4D)	TCTGCACTTCGGCGGTTACATG	CTGAGAGCAGATTCATTGGCA

RT-qPCR target	Sample	Ct value
eef1b2 (Fig. S1D)	*tnnt2a*$^{+/+}$ 1	20.23
eef1b2 (Fig. S1D)	*tnnt2a*$^{+/+}$ 2	21.26
eef1b2 (Fig. S1D)	*tnnt2a*$^{+/+}$ 3	20.32
eef1b2 (Fig. S1D)	*tnnt2a*$^{flox/flox}$ 1	20.22
eef1b2 (Fig. S1D)	*tnnt2a*$^{flox/flox}$ 2	20.08
eef1b2 (Fig. S1D)	*tnnt2a*$^{flox/flox}$ 3	20.16
eef1b2 (Fig. S1D)	*tnnt2a*$^{flox/flox}$ + *Cre* mRNA 1	18.03
eef1b2 (Fig. S1D)	*tnnt2a*$^{flox/flox}$ + *Cre* mRNA 2	17.98
eef1b2 (Fig. S1D)	*tnnt2a*$^{flox/flox}$ + *Cre* mRNA 3	18.20
tnnt2a (Fig. S1D)	*tnnt2a*$^{+/+}$ 1	28.03
tnnt2a (Fig. S1D)	*tnnt2a*$^{+/+}$ 2	28.22
tnnt2a (Fig. S1D)	*tnnt2a*$^{+/+}$ 3	28.01
tnnt2a (Fig. S1D)	*tnnt2a*$^{flox/flox}$ 1	27.77
tnnt2a (Fig. S1D)	*tnnt2a*$^{flox/flox}$ 2	27.60
tnnt2a (Fig. S1D)	*tnnt2a*$^{flox/flox}$ 3	27.57
tnnt2a (Fig. S1D)	*tnnt2a*$^{flox/flox}$ + *Cre* mRNA 1	26.79
tnnt2a (Fig. S1D)	*tnnt2a*$^{flox/flox}$ + *Cre* mRNA 2	26.68
tnnt2a (Fig. S1D)	*tnnt2a*$^{flox/flox}$ + *Cre* mRNA 3	26.71
eef1b2 (Fig. S4D)	*tnnt2a*$^{+/+}$ 1	21.34
eef1b2 (Fig. S4D)	*tnnt2a*$^{+/+}$ 2	21.44
eef1b2 (Fig. S4D)	*tnnt2a*$^{+/+}$ 3	22.10
eef1b2 (Fig. S4D)	*tnnt2a*$^{bns511/bns511}$ 1	19.02
eef1b2 (Fig. S4D)	*tnnt2a*$^{bns511/bns511}$ 2	18.12
eef1b2 (Fig. S4D)	*tnnt2a*$^{bns511/bns511}$ 3	18.17
eef1b2 (Fig. S4D)	*tnnt2a*$^{bns513/bns513}$ 1	21.09
eef1b2 (Fig. S4D)	*tnnt2a*$^{bns513/bns513}$ 2	20.52
eef1b2 (Fig. S4D)	*tnnt2a*$^{bns513/bns513}$ 3	20.29
tnnt2a (Fig. S4D)	*tnnt2a*$^{+/+}$ 1	25.98
tnnt2a (Fig. S4D)	*tnnt2a*$^{+/+}$ 2	25.88
tnnt2a (Fig. S4D)	*tnnt2a*$^{+/+}$ 3	26.05
tnnt2a (Fig. S4D)	*tnnt2a*$^{bns511/bns511}$ 1	25.41
tnnt2a (Fig. S4D)	*tnnt2a*$^{bns511/bns511}$ 2	25.19
tnnt2a (Fig. S4D)	*tnnt2a*$^{bns511/bns511}$ 3	25.06
tnnt2a (Fig. S4D)	*tnnt2a*$^{bns513/bns513}$ 1	26.17
tnnt2a (Fig. S4D)	*tnnt2a*$^{bns513/bns513}$ 2	25.73
tnnt2a (Fig. S4D)	*tnnt2a*$^{bns513/bns513}$ 3	25.68

SI Methods

CRISPR/Cas9 knock-in

CRISPR/Cas9 knock-in was performed according to published protocols for the creation of the *tnnt2a*^{flox} allele (using a ssODN donor and 49-21bp asymmetric homology arms) (1) and the *tnnt2a-eGFP* fusion *tnnt2a*^{bns511} and *tnnt2a*^{bns513} alleles (using a pGTag donor vector and 48 bp symmetric homology arms) (2). The concatemer presence in *tnnt2a*^{bns511} was confirmed using pGTag backbone-specific PCR. The CRISPR site and primer binding sites used to insert the second LOXP site in the *tnnt2a*^{mn0031Gt} background can be found using the GenBank ID AF512525.1, as the reference sequence for *tnnt2a* (ENSDARG00000020610, transcript *tnnt2a-204*) does not contain them. The sequences of the target CRISPR sites used for the knock-in, the donor oligonucleotide used for the LOXP insertion, as well as the primers used to assess the integration and genotype of the created lines are available in Supplementary table S1.

Zebrafish strains and genotyping

The following published lines were used for this study: *tnnt2a*^{tc300b} (3), *tnnt2a*^{mn0031Gt} (or *Gt(GBT-R14)*) (4), *Tg(myl7:Cre)*^{sd55} (5), *Tg(cryaa:DsRed,-5,1myl7:Cre-ERT2)*^{pd10} (6), *Tg(myh6:Cre-ERT2)*^{sd20} (7), *Tg(hsp70l:Cre)*^{zdf13} (8), *Tg(-3,5ubb:LOXP-eGFP-LOXP-mCherry)*^{cz1701} (9), *Tg(myl7:LIFEACT-Tomato)*^{bns141} (10), *Tg(hsp70l:zGRAD-IRES-h2a-TagBFP)*^{sk93} (11) and *Tg(actb2:LOXP-mCherry-LOXP-zGRAD)*^{hsc185} (12). The following transgenic lines were newly generated: *Pt(tnnt2a:LOXP-tnnt2a-LOXP)*^{bns625} (or *tnnt2a*^{flox}), *Pt(tnnt2a:tnnt2a-eGFP)*^{bns511} (or *tnnt2a*^{bns511}), *Pt(tnnt2a:tnnt2a-eGFP)*^{bns513} (or *tnnt2a*^{bns513}), *Tg(myl7:zGRAD-P2A-TagBFP)*^{bns626}, *Tg(myl7:split-tPT2A-zGRAD-tPT2a-TagBFP)*^{bns704} (or *Tg(myl7:split-zGRAD)*^{bns704}), *Tg(myl7:LOXP-stop-LOXP-zGRAD-P2A-TagBFP-CAAX,gcry:eGFP)*^{bns672}, and *Tg(myh7:zfCre-ERT2)*^{bns594}.

For the experiments with *tnnt2a*^{tc300b} mutants, we intercrossed heterozygous parents, identified the homozygous mutants based on the *silent heart* phenotype at 24 hpf (3) and genotyped the siblings using a mutant-specific PCR.

For the experiments with *tnnt2a*^{flox} transgenics, we intercrossed heterozygous parents and genotyped the progeny using wild-type and transgene-specific PCR. The primer that binds the transgene is located in a region of *tnnt2a*^{mn0031Gt} that remains after Cre-removal of the cassette and that contains a transposase integration, which likely happened after F0 injection during the establishment of the line (4).

For the experiments with *tnnt2a*$^{mn0031Gt/+}$ and *tnnt2a*$^{mn0031Gt/bns511}$ or *tnnt2a*$^{mn0031Gt/bns513}$ transgenics, we intercrossed heterozygous parents and identified the *tnnt2a*^{mn0031Gt} and *tnnt2a*$^{bns511\ or\ bns513}$ alleles based on mRFP (4) and eGFP expression in the heart, respectively.

For the experiments with *tnnt2a*^{bns513} homozygous transgenics, we intercrossed heterozygous parents and genotyped the progeny using wild-type and transgene-specific PCR.

For the experiments with the *myl7:Cre*^{sd55}, *myh6:Cre-ERT2*^{pd10}, *myh7:zfCre-ERT2*^{bns594}, and *hsp70l:Cre*^{zdf13} transgenes, we outcrossed heterozygous parents and performed a *Cre* specific PCR on the progeny when the recombination was not visible using *-3,5ubb:LOXP-eGFP-LOXP-mCherry*^{cz1701} recombination.

For the experiments with the *cryaa:DsRed,-5,1myl7:Cre-ERT2*^{pd10} and *-0.8myl7:LOXP-stop-LOXP-zGRAD-P2A-TagBFP-CAAX,gcry:eGFP*^{bns672} transgenes, we outcrossed heterozygous parents and used the DsRed and eGFP eye markers to identify the heterozygous embryos at 56 hpf after unmounting the embryos following imaging.

For the experiments with the *myl7:LIFEACT-Tomato*^{bns141}, *actb2:LOXP-mCherry-LOXP-zGRAD*^{hsc185}, *myl7:zGRAD-P2A-TagBFP*^{bns626}, and *myl7:split-zGRAD* transgenes, we outcrossed heterozygous parents and used Tomato, mCherry, and TagBFP expression to identify them at 32 hpf.

For the experiments with the *hsp70l:zGRAD-IRES-h2a-TagBFP*^{sk93} transgene, as the TagBFP is reported not to be detectable (11), we outcrossed heterozygous parents and identified them using a *zGRAD* specific PCR.
PCR amplifications were performed with KAPA2G Fast Ready Mix (Sigma 2GFRMKB) with the following cycling conditions: Initial denaturation 3 min 95 °C; 10 cycles (15 s 95 °C, 30 s 65 °C first to 55 °C last cycle, 30 s 72 °C); 30 (17 for *tnnt2a*^{tc300b} mutants) cycles (15 s 95 °C, 30 s 55 °C, 30 s 72 °C); final extension 1 min 72 °C. All primers used to genotype the alleles described in this study are listed in Supplementary table S1.

Plasmid generation and injections

The tol2 plasmids used to generate the *Tg(myl7:zGRAD-P2A-TagBFP)*^{bns626}, *Tg(myl7:split-zGRAD)*^{bns704}, *Tg(myl7:LOXP-stop-LOXP-zGRAD-P2A-TagBFP-CAAX,gcry:eGFP)*^{bns672}, and *Tg(myh7:zfCre-ERT2)*^{bns594} lines, and to carry out the transient analysis shown in Fig. S9 (*myl7/myh7/myh6:split-zGRAD*), were assembled using *in vivo* cloning (13) from published *myl7* (14), *myh7 (15), and myh6* (7) promoters, and *zGRAD* (11), *TagBFP* (16), *Cre-zf1 (17)*, *ERT2* (18), *cpFRB2-FKBP* (19), and *tPT2A* (20) sequences.
pCS2-*Cre.zf1* was a gift from Harold Burgess (Addgene plasmid # 61391)(17) which we linearized using NotI, and *Cre* mRNA was *in vitro* synthesized using the mMessage mMACHINE SP6 transcription kit (Invitrogen AM1340). We injected 6 pg of *Cre* mRNA for LOXP sites recombination, and 15 pg of plasmid DNA together with 15 pg of transposase mRNA for transgenic line establishment and transient experiments. All reagents were injected at the one-cell stage at a volume of 1 nl together with 0,2% phenol red.

PCR and RT-qPCR analysis

PCR reactions on genomic DNA and cDNA to visualize *tnnt2a*^{flox} recombination (Fig. S1B-C) and concatemer removal in *tnnt2a*^{bns513} (Fig. S4B-C) were performed on single embryos using KAPA2G Fast Ready Mix (Sigma 2GFRMKB) (Fig. S1B,S2C) and PrimeSTAR Max DNA Polymerase (Takara) (Fig. S1C,S4B-C). RT-qPCR analysis was performed on pools of 5 embryos and the results represent biological triplicates with two technical duplicates per biological replicate. *eef1b2* was used as a reference gene for all RT-qPCR conditions and fold changes were calculated using the 2−ΔΔCt method. RNA was isolated using TRIzol extraction and reverse transcription was performed using Maxima First Strand cDNA synthesis (Thermo Fisher) for PCR on cDNA and RT-qPCR experiments. Embryos were PCR genotyped prior to RNA extraction for PCR on cDNA and RT-qPCR experiments by cutting a piece of the tail, which doesn't express *tnnt2a* mRNA (21). All Ct values and primers are listed in Supplementary table S1.

RNA *in situ* hybridizations

Whole-mount RNA in situ hybridizations were performed according to standard protocols (22). The *in situ* probe for *tnnt2a* was synthesized directly from a PCR product, performed on wild-type AB cDNA at 78 hpf (23), using an incorporated T7 promoter (Supplementary table S1). *In situ* hybridizations were imaged using a SMZ25 stereomicroscope (Nikon) with a 2x/0.3 objective.

Fluorescence imaging of hearts

Live cardiac imaging was performed on embryos anesthetized in 0,0175% tricaine and cardiac contractions were stopped using 25 mM BDM (Sigma B0753) (Fig. 6B-D,S7B,S8A-D',S9A-C). Alternatively, embryos were fixed in 4% PFA for 2 h at room temperature, the yolk removed to access the heart, and mounted directly for imaging (Fig. S2D-G,S5B-D) or additionally stained with Phalloidin (1:500, Invitrogen A12380) (Fig. 3E,4E-G). Images were acquired using an LSM 700 confocal laser scanning microscope (Zeiss) using a 40x objective with a pixel width of 0,52 µm and a z-step of 2 µm.

Imaging and representation of blood flow motion

We performed brightfield imaging of whole embryos and larvae for 5 sec at 16 frames/sec with a pixel width of 3,24 µm using a SMZ25 stereomicroscope (Nikon) with a 2x/0.3 objective. We applied a standard deviation z-projection to the 10 first frames using ImageJ, which highlights the variations in pixel intensity during this time window, such as red blood cells moving through the vessels. We colored the obtained projection using the "Red Hot" lookup table. We then merged the projection channel with a snapshot image of the same embryo.

Live heart imaging and kymograph representation

Hearts were imaged while beating for 4,2 sec at 240 frames/sec with a pixel width of 0,52 µm using an inverted Cell Observer Spinning Disk microscope with a 25x objective. We applied a variance 3D filter to the obtained movie with a radius of x=1,y=1,z=10 using ImageJ and colored the result using the "Red Hot" lookup table. We then merged a kymograph of the brightfield movie and the variance movie, centered horizontally on the ventricle or atrium. The whole heart brightfield image shown represents one frame at atrial diastole.

Western blot analysis

We dissected the upper body without the yolk of 12 embryos per replicate at 48 hpf using micro-syringes and kept them dry at -80°C. Embryos were crushed with a pestle in 48 µl of 4x Laemmli Sample Buffer (Bio-Rad 1610747) supplemented with 2-mercaptoethanol. Samples were then heated at 70°C for 10 min, the volume equivalent to six embryos per well was loaded on a 4-12% Bis-Tris NuPAGE™ gel (Invitrogen NP0335), and ran at 180 V for 1 h. Protein transfer was done at 4°C for 1 h at 110 V onto a 0.2 µm nitrocellulose membrane (Invitrogen LC2000). The membrane was blocked in 5% milk-PBT (PBS+0.1% Tween 20) for 40 min and incubated overnight at 4 °C with rabbit anti-GFP (1:1000, Invitrogen A11122) or mouse anti-β-actin (1:1000, Sigma A5441) primary antibodies in PBT. Anti-mouse (1:4000, Abcam ab97023) and anti-rabbit (1:4000, Cell Signaling 7074) HRP-linked secondary antibodies were used and incubated 1 h at room temperature in 5% milk-PBT. Chemiluminescent detection was performed using the SuperSignal™ West Pico PLUS chemiluminescent substrate kit (ThermoFisher 34577) on a Biorad ChemiDoc™ MP Imaging System.

Drug and heat shock treatments

4-Hydroxytamoxifen (Sigma H7904) stock was prepared at 25 mM in DMSO and used at 5 µM to treat embryos between 8 and 56 hpf (Fig. 2,S2) or between 8 and 48 hpf (Fig. S6), with a media and drug refreshment at 32 hpf. Rapamycin (Sigma R0395) stock was prepared at 1 mM in DMSO and used at 2 µM to treat embryos between 48 and 72 hpf. We selected 2 µM as a working concentration as it induces only mild developmental defects even when used for 5 days (24). Control embryos were treated with the same volume of DMSO as the drug-treated ones. Drug treatment was carried out in the dark. We added 1-phenyl-2-thiourea (Sigma P7629) at 30 mg/l to prevent embryonic pigmentation. Heat shock was performed by placing the embryos in pre-heated media at 39°C for 1 h two times in a row, spaced by 1 h at 28°C (Fig. S3A), starting at 6 (Fig. S3B,D,D'), 24 (Fig. S3E,E'), or 32 (Fig. S8B) hpf.

Heart cells dissociation and single-cell RNA sequencing

We incrossed *tnnt2a*$^{mn0031Gt/+}$ fish to generate *tnnt2a*$^{+/+}$ and *tnnt2a*$^{mn0031Gt/mn0031Gt}$ progeny (Fig. 5A). We intercrossed *tnnt2a*$^{mn0031Gt/+}$ with *tnnt2a*$^{bns513/+}$; *myl7:zGRAD-P2A-TagBFP*$^{+/-}$ to generate *tnnt2a*$^{mn0031Gt/bns513}$; *myl7:zGRAD-P2A-TagBFP*$^{+/-}$ progeny (Fig. 5A). We dissected 100 hearts per condition at 72 hpf using micro-syringes in cold DMEM (ThermoFisher 88281)+10% FBS (Sigma F2442). We pelleted the hearts in 1,5 ml eppendorf tubes by centrifugation at 3000 rpm for 5 min at 4°C and replaced the media to cold 1X HBSS (ThermoFisher 88281). We repeated the previous step to get rid of the FBS, which can

disrupt the dissociation enzyme activity, and removed the excess media. We prepared the dissociation enzyme 1 (with papain) and enzyme 2 (with thermolysin) according to manufacturer's protocol (ThermoFisher 88281). We then mixed 150 µl of enzyme 1 with 15 µl of enzyme 2, and added 50 µl of the mix to each of the 3 conditions. We kept the tubes in a 300 rpm shaker at 37°C for 30 min, quenched the reaction by adding 1 ml of DMEM-10% FBS, and finished to break up the tissues by pipetting up and down. We pelleted the heart cells by centrifugation at 3000 rpm for 5 min at 4°C and replaced the media to PBS without Calcium or Magnesium (Lonza 17-516F)+0.04% BSA.
The cell suspensions were counted with a Moxi cell counter and diluted according to manufacturer's protocol to obtain 10.000 single cell data points per sample. Each sample was run separately on a lane in a Chromium controller with Chromium Next GEM Single Cell 3' Reagent Kits v3,1 (10xGenomics). Single cell RNAseq library preparation was done using a standard protocol. Sequencing was done on a Nextseq2000.

Single-cell transcriptome analysis

Sequenced raw reads were aligned against the zebrafish genome (DanRer11) and counted by StarSolo (25). Counts per gene and cell identifiers were stored in an Annotated Data Format (26) for all further analysis steps, holding initially 7175 cells × 26339 genes. Next, cells were analyzed via the scanpy framework (27). Preprocessed counts were used to calculate quality metrics and estimate cell quality taking into account ribosomal content, mitochondrial content, number of genes, and total read count. Doublets were removed by the scrublet tool (28). In summary, we reduced the cell number to 5432 considered high-quality cells with a total of 25415 genes expressed after mitochondrial and ribosomal gene exclusion. Following QC, raw counts per cell were normalized to the median count of all cells and transformed into log space to stabilize the variance. Batch correction on samples was done by using harmony (29). After calculating PCA followed by neighbors calculation (15 nearest neighbors), we generated low-dimensional UMAP embedding (30) by using a minimum distance of 0,4 and a spread of 2,5. Clustering was done by leiden with a resolution of 0,9. From the final UMAP, we manually removed four clusters annotated as leftover doublets and red blood cells, finally ending up with 5120 cells. Final data visualization was done using the CellxGene package (doi:10.5281/zenodo.3235020).

SI References

1. L. Burg *et al.*, Conditional mutagenesis by oligonucleotide-mediated integration of loxP sites in zebrafish. *PLoS Genet* **14**, e1007754 (2018).
2. J. M. Welker *et al.*, GeneWeld: Efficient Targeted Integration Directed by Short Homology in Zebrafish. *Bio Protoc* **11**, e4100 (2021).
3. A. J. Sehnert *et al.*, Cardiac troponin T is essential in sarcomere assembly and cardiac contractility. *Nat Genet* **31**, 106-110 (2002).
4. K. J. Clark *et al.*, In vivo protein trapping produces a functional expression codex of the vertebrate proteome. *Nat Methods* **8**, 506-515 (2011).
5. P. Han *et al.*, Coordinating cardiomyocyte interactions to direct ventricular chamber morphogenesis. *Nature* **534**, 700-704 (2016).
6. K. Kikuchi *et al.*, Primary contribution to zebrafish heart regeneration by gata4(+) cardiomyocytes. *Nature* **464**, 601-605 (2010).
7. R. Zhang *et al.*, In vivo cardiac reprogramming contributes to zebrafish heart regeneration. *Nature* **498**, 497-501 (2013).
8. H. Feng *et al.*, Heat-shock induction of T-cell lymphoma/leukaemia in conditional Cre/lox-regulated transgenic zebrafish. *Br J Haematol* **138**, 169-175 (2007).
9. C. Mosimann *et al.*, Ubiquitous transgene expression and Cre-based recombination driven by the ubiquitin promoter in zebrafish. *Development* **138**, 169-177 (2011).

10. R. Fukuda *et al.*, Proteolysis regulates cardiomyocyte maturation and tissue integration. *Nat Commun* **8**, 14495 (2017).
11. N. Yamaguchi, T. Colak-Champollion, H. Knaut, zGrad is a nanobody-based degron system that inactivates proteins in zebrafish. *eLife* **8** (2019).
12. M. Jussila, C. W. Boswell, N. W. Griffiths, P. G. Pumputis, B. Ciruna, Live imaging and conditional disruption of native PCP activity using endogenously tagged zebrafish sfGFP-Vangl2. *Nat Commun* **13**, 5598 (2022).
13. J. F. Watson, J. Garcia-Nafria, In vivo DNA assembly using common laboratory bacteria: A re-emerging tool to simplify molecular cloning. *J Biol Chem* **294**, 15271-15281 (2019).
14. C. J. Huang, C. T. Tu, C. D. Hsiao, F. J. Hsieh, H. J. Tsai, Germ-line transmission of a myocardium-specific GFP transgene reveals critical regulatory elements in the cardiac myosin light chain 2 promoter of zebrafish. *Dev Dyn* **228**, 30-40 (2003).
15. R. Zhang, X. Xu, Transient and transgenic analysis of the zebrafish ventricular myosin heavy chain (vmhc) promoter: an inhibitory mechanism of ventricle-specific gene expression. *Dev Dyn* **238**, 1564-1573 (2009).
16. O. M. Subach *et al.*, Conversion of red fluorescent protein into a bright blue probe. *Chemistry & biology* **15**, 1116-1124 (2008).
17. E. J. Horstick *et al.*, Increased functional protein expression using nucleotide sequence features enriched in highly expressed genes in zebrafish. *Nucleic Acids Res* **43**, e48 (2015).
18. D. Metzger, J. Clifford, H. Chiba, P. Chambon, Conditional site-specific recombination in mammalian cells using a ligand-dependent chimeric Cre recombinase. *Proc Natl Acad Sci U S A* **92**, 6991-6995 (1995).
19. Y. T. Lee, L. He, Y. Zhou, Expanding the Chemogenetic Toolbox by Circular Permutation. *J Mol Biol* **432**, 3127-3136 (2020).
20. Z. Liu *et al.*, Systematic comparison of 2A peptides for cloning multi-genes in a polycistronic vector. *Sci Rep* **7**, 2193 (2017).
21. C. D. Hsiao, W. Y. Tsai, L. S. Horng, H. J. Tsai, Molecular structure and developmental expression of three muscle-type troponin T genes in zebrafish. *Dev Dyn* **227**, 266-279 (2003).
22. C. Thisse, B. Thisse, High-resolution in situ hybridization to whole-mount zebrafish embryos. *Nat Protoc* **3**, 59-69 (2008).
23. T. Juan *et al.*, Multiple pkd and piezo gene family members are required for atrioventricular valve formation. *Nat Commun* **14**, 214 (2023).
24. C. Sucularli *et al.*, Functionally conserved effects of rapamycin exposure on zebrafish. *Mol Med Rep* **13**, 4421-4430 (2016).
25. A. Dobin *et al.*, STAR: ultrafast universal RNA-seq aligner. *Bioinformatics* **29**, 15-21 (2013).
26. I. Virshup, S. Rybakov, F. J. Theis, P. Angerer, F. A. Wolf, anndata: Annotated data. *bioRxiv* 10.1101/2021.12.16.473007, 2021.2012.2016.473007 (2021).
27. F. A. Wolf, P. Angerer, F. J. Theis, SCANPY: large-scale single-cell gene expression data analysis. *Genome Biol* **19**, 15 (2018).
28. S. L. Wolock, R. Lopez, A. M. Klein, Scrublet: Computational Identification of Cell Doublets in Single-Cell Transcriptomic Data. *Cell Syst* **8**, 281-291 e289 (2019).
29. V. A. Traag, L. Waltman, N. J. van Eck, From Louvain to Leiden: guaranteeing well-connected communities. *Scientific reports* **9**, 5233 (2019).
30. L. McInnes, J. Healy, J. Melville, UMAP: Uniform Manifold Approximation and Projection for Dimension Reduction. http://dx.doi.org/10.48550/arXiv.1802.03426.
